# Supplementary material for: Sequencing smart: De novo sequencing and assembly approaches for a non-model mammal
Source: Gigascience. 2020 May 12;9(5):giaa045. doi: 10.1093/gigascience/giaa045 (PMC7216774; doi:10.1093/gigascience/giaa045)
Supplement: giaa045_GIGA-D-19-00279_Revision_2 [file giaa045_giga-d-19-00279_revision_2.pdf]

GigaScience

Sequencing smart: De novo sequencing and assembly approaches for a non-model mammal.

--Manuscript Draft--

**Manuscript Number:**	GIGA-D-19-00279R2
**Full Title:**	Sequencing smart: De novo sequencing and assembly approaches for a non-model mammal.
**Article Type:**	Research
**Funding Information:**	Biotechnology and Biological Sciences Research Council (BBS/E/T/000PR9817) — Not applicable<br>Biotechnology and Biological Sciences Research Council (BB/CCG1720/1) — Not applicable
**Abstract:**	Background<br>Whilst much sequencing effort has focused on key mammalian model organisms such as mouse and human, little is known about the relationship between genome sequencing techniques for non-model mammals and genome assembly quality. This is especially relevant to non-model mammals, where the samples to be sequenced are often degraded and low quality. A key aspect when planning a genome project is the choice of sequencing data to generate. This decision is driven by several factors, including the biological questions being asked, the quality of DNA available, and the availability of funds. Cutting-edge sequencing technologies now make it possible to achieve highly contiguous, chromosome-level genome assemblies, but relies on good quality high-molecular-weight DNA. The funds to generate and combine these data are often only available within large consortiums and sequencing initiatives, and are often not affordable for many independent research groups. For many researchers, value-for-money is a key factor when considering the generation of genomic sequencing data. Here we use a range of different genomic technologies generated from a roadkill European Polecat (Mustela putorius) to assess various assembly techniques on this low-quality sample. We evaluated different approaches for de novo assemblies and discuss their value in relation to biological analyses.<br><br>Results<br>Generally, assemblies containing more data types achieved better scores in our ranking system. However, when accounting for misassemblies, this was not always the case for Bionano and low-coverage 10x Genomics (for scaffolding only). We also find that the extra cost associated with combining multiple data types is not necessarily associated with better genome assemblies.<br><br>Conclusions<br>The high degree of variability between each de novo assembly method (assessed from the seven key metrics) highlights the importance of carefully devising the sequencing strategy to be able to carry out the desired analysis. Adding more data to genome assemblies does not always results in better assemblies so it is important to understand the nuances of genomic data integration explained here, in order to obtain cost-effective value-for-money when sequencing genomes.
**Corresponding Author:**	Graham John Etherington, BSc, PhD<br>Earlham Institute<br>Norwich, Norfolk UNITED KINGDOM
**Corresponding Author Secondary Information:**	
**Corresponding Author's Institution:**	Earlham Institute
**Corresponding Author's Secondary Institution:**	
**First Author:**	Graham John Etherington, BSc, PhD
**First Author Secondary Information:**	

Powered by Editorial Manager® and ProduXion Manager® from Aries Systems Corporation

**Order of Authors:**	Graham John Etherington, BSc, PhD
	Darren Heavens
	David Baker
	Ashleigh Lister
	Rose McNelly
	Gonzalo Garcia
	Bernardo Clavijo
	Iain Macaulay
	Wilfried Haerty
	Federica Di Palma
**Order of Authors Secondary Information:**	
**Response to Reviewers:**	See attached document 'Response2.docx'
**Additional Information:**	
**Question**	**Response**
Are you submitting this manuscript to a special series or article collection?	No
**Experimental design and statistics**<br><br>Full details of the experimental design and statistical methods used should be given in the Methods section, as detailed in our Minimum Standards Reporting Checklist. Information essential to interpreting the data presented should be made available in the figure legends.<br><br>Have you included all the information requested in your manuscript?	Yes
**Resources**<br><br>A description of all resources used, including antibodies, cell lines, animals and software tools, with enough information to allow them to be uniquely identified, should be included in the Methods section. Authors are strongly encouraged to cite Research Resource Identifiers (RRIDs) for antibodies, model organisms and tools, where possible.<br><br>Have you included the information requested as detailed in our Minimum Standards Reporting Checklist?	Yes

Powered by Editorial Manager® and ProduXion Manager® from Aries Systems Corporation

**Availability of data and materials**<br><br>All datasets and code on which the conclusions of the paper rely must be either included in your submission or deposited in publicly available repositories (where available and ethically appropriate), referencing such data using a unique identifier in the references and in the “Availability of Data and Materials” section of your manuscript.<br><br>Have you have met the above requirement as detailed in our Minimum Standards Reporting Checklist?	Yes

Powered by Editorial Manager® and ProduXion Manager® from Aries Systems Corporation

Manuscript

Click here to access/download;Manuscript;Manuscript_v3.docx

Sequencing smart: *De novo* sequencing and assembly approaches for a non-model mammal.

Graham J Etherington*, Darren Heavens, David Baker, Ashleigh Lister, Rose McNelly, Gonzalo Garcia, Bernardo Clavijo, Iain Macaulay, Wilfried Haerty, Federica Di Palma*.

The Earlham Institute, Norwich Research Park, Norwich, NR4 7UZ, United Kingdom

*Corresponding authors – graham.etherington@earlham.ac.uk, Federica.di-palma@earlham.ac.uk

Graham J Etherington: 0000-0002-5003-1425; Darren Heavens: 0000-0001-5418-7868; Bernardo Clavijo: 0000-0002-7597-2774; Wilfried Haerty: 0000-0003-0111-191X; Federica Di Palma: 0000-0002-4394-0102

Keywords: polecat, vertebrate, non-model organism, Illumina, chromium, Bionano, assembly, sequencing.

Abstract

Background

Whilst much sequencing effort has focused on key mammalian model organisms such as mouse and human, little is known about the relationship between genome sequencing techniques for non-model mammals and genome assembly quality. This is especially relevant to non-model mammals, where the samples to be sequenced are often degraded and low quality. A key aspect when planning a genome project is the choice of sequencing data to generate. This decision is driven by several factors, including the biological questions being asked, the quality of DNA available, and the availability of funds. Cutting-edge sequencing technologies now make it possible to achieve highly contiguous, chromosome-level genome assemblies, but relies on good quality high-molecular-weight DNA. The funds to generate and combine these data are often only available within large consortiums and sequencing initiatives, and are often not affordable for many independent research groups. For many researchers, value-for-money is a key factor when considering the generation of genomic sequencing data. Here we use a range of different genomic technologies generated from a roadkill European Polecat (*Mustela putorius*) to assess various assembly techniques on this low-quality sample. We evaluated different approaches for *de novo* assemblies and discuss their value in relation to biological analyses.

Results

Generally, assemblies containing more data types achieved better scores in our ranking system. However, when accounting for misassemblies, this was not always the case for Bionano and low-coverage 10x Genomics (for scaffolding only). We also find that the extra cost associated with combining multiple data types is not necessarily associated with better genome assemblies.

Conclusions

The high degree of variability between each *de novo* assembly method (assessed from the seven key metrics) highlights the importance of carefully devising the sequencing strategy to be able to carry out the desired analysis. Adding more data to genome assemblies does not always results in better assemblies so it is important to understand the nuances of genomic data integration explained here, in order to obtain cost-effective value-for-money when sequencing genomes.

Introduction

Starting in 1990, the Human Genome Project used low-throughput, high-cost Sanger sequencing platforms to create the first draft human genome at a cost of USD $300 million. Fast-forward 19 years and the cost of sequencing a human genome has dropped to around USD $1,000. Short-read technologies producing high-throughput, low per-base cost next-generation sequencing (NGS) means that genomics is no longer restricted to large sequencing consortiums and has opened up the field to even the smallest of research groups. The recently formed Vertebrate Genomes Project (VGP) aims to produce near-gapless, chromosome-scale phased genome assemblies for around 66,000 extant vertebrate species [1]. The assembly pipeline consists of 60x coverage Pacific Biosciences (PacBio) long read sequencing, followed by 10x Genomics linked reads, Bionano optical mapping and Arima Genomics' Hi-C profiles. These long-read technologies provide highly contiguous genome assemblies. Similar consortiums and sequencing initiatives have been formed to sequence a range of target organisms such as Bat1K, Bird10K, Oz Mammals Genomics, and Earth BioGenome Project (including Darwin UK Tree of Life, Colombia EBP, etc.) [2]. Although such efforts make it possible to achieve highly-contiguous, chromosome level genome assemblies, the

cost of generating this amount of data and assembling them is considerable and often only within reach of a few of these consortiums. It is important for smaller independent research groups or initiatives to consider value-for-money against biological questions as a key factor when planning the generation of genomic sequencing data.

Non-model organisms have the potential to provide new knowledge related to phenotypic and genotypic variation. Through comparative genomics, it is possible to identify how different organisms are related to each other, how they adapt to novel environments, or the genetic basis underlying novel phenotypes. These new findings can be applied to further research, such as in the biomedical and food industries through breeding programs with the development of marker assisted selection and in conservation biology [3-12].
De novo assembly of endangered species, followed by low-coverage population-level sequencing provides unprecedented information about the amount of genetic diversity within populations, past and ongoing gene flow between different populations, and the level of inbreeding in small populations [13-17].

However, there are a number of difficulties when working with non-model mammals. Firstly, the genome size is not always known, hampering the assessment of the completeness of the ‘assembled’ genome and of the sequencing depth. Additionally, the availability and quality of the samples used for sequencing non-model organisms is often substandard. Tissue and blood samples are often obtained from wild populations and may need to be acquired from remote locations, delaying the time between collection and DNA extraction. Another common issue relates to samples which may have been stored in collections such as museums, zoos and tissue collections and subjected to a number of different preservation methods such as freezing, storage in ethanol, FFPE, etc. Many current sequencing technologies rely on high-

molecular-weight DNA with varying optimum molecule lengths (e.g. PacBio HiFi reads 15-20kb, Bionano > 150kb, and 10x Genomics > 50kb). Degraded DNA, as is commonly observed in samples from wild populations is usually sub-optimal for use in many advanced sequencing methods. It is therefore difficult, or sometimes impossible, to leverage the full application of these technologies.

Many samples from non-model organisms originate from wild populations that are highly heterozygous leading to numerous challenges during the assembly step. Allelic differences in a diploid genome generates branches and bubbles in the assembly graph [18]. Even though most graph-based assemblers have functions to search for and remove these structures, high density variation can still make assembly of heterozygous organisms challenging. Conversely, high levels of homozygosity, characteristic of endangered (and typically inbred) species, hamper the efforts of creating phased genome assemblies, since the ability to phase haplotypes is dependent on linked sequences spanning polymorphisms. Additionally, non-model organisms vary in their ploidy, chromosome number, repeat content, sequence composition and GC content, adding further confounding factors to genome assembly.

The European Polecat (*Mustela putorius*, NCBI:txid9668) is a medium-sized carnivore found across Europe and the Middle East. It is purported to be the ancestral species of the domestic ferret (*M. p. furo*) [19]. Across most of mainland Europe the polecat is in widespread decline [20]. In the United Kingdom, the European Polecat has a chequered history. Persecuted to the verge of extinction in the early-1900's, when it was confined to unmanaged forests in central Wales, it has since seen a population increase and is now found throughout Wales and across much of central, south-western and eastern England [21].

Here, a road-kill sample of European Polecat from the Vincent Wildlife Trust collection (VWT 693) was used to assess short-read and long-range *de novo* sequencing strategies for non-model mammals. Comparisons between combinations of PCR-free Illumina libraries, Nextera long-mate-pair (LMP) libraries, 10x Genomics Chromium libraries and Bionano optical maps are made to assess optimum sequencing and assembly strategies.

Sequencing technologies

Short-read sequencing

The market-leader in short-read high-throughput NGS is Illumina [22]. Machines produce read-lengths of 100 bps and above and a single Illumina Novaseq run is currently capable of generating 3,000 Gbps of read data. An advantage to Illumina sequencing is the generation of paired-end (PE) reads, in which the sequence from both ends of each DNA molecule is synthesised. As the input molecules are of an approximate known length, the acquisition of PE data provides a greater amount of information. Additionally, using a PCR-free library preparation removes bias in genomic coverage previously incorporated by a PCR amplification step in older library preparation procedures [23]. Although requiring input DNA of a degree of magnitude greater than PCR-amplified libraries, PCR-free libraries are expected to capture unbiased coverage of the genome, usually reflected by an increased size of the assembly and less duplication in single-copy regions of the genome compared with PCR-amplified libraries [24]. They also provide superior coverage in GC-rich regions of the genome, enabling access to regions that were previously difficult to sequence [25]. PCR-free Illumina sequencing requires a minimum of 2 μg of genomic DNA (gDNA) at a minimum concentration of 35 ng/μl in 60 μl.

Long Mate Pair sequencing.

Long DNA fragments up to around 40 kb can be sequenced to provide PE reads that bridge long repeats, thus producing longer contiguous genome assemblies as well as characterising structural variants. Under the Nextera LMP protocol [26], a transposase enzyme attaches 19-bp biotinylated adaptors to both ends of each long DNA fragment. The DNA is then circularized, where the biotinylated ends become joined. The circularized DNA is then fragmented and biotin enrichment is used to process the fragments containing the adaptors that mark the junction. During sequencing, reads are produced from both ends of a fragment, resulting in inward-facing reads that read toward and through the adaptors. 12 libraries covering a wide range of jump sizes can be constructed using this protocol, thus ensuring production of the best LMP libraries from a given DNA sample. For Illumina Nextera LMP sequencing the Nextclip tool can then be used to trim adaptors and de-duplicate reads [27]. Nextera LMP sequencing requires a minimum of 4 μg of gDNA for the 12 libraries, at a minimum concentration of 30 ng/μl in 300 μl.

10x Genomics

The Chromium system from 10x Genomics uses oil emulsion and multiple displacement amplification (MDA) to ligate short molecular barcodes to reads from each fragment of DNA, followed by PE Illumina sequencing [28]. Each fragment receives its own unique barcode and hence reads with the same barcodes represents clusters of reads from the same region in the genome. These ‘linked-reads’ provide the long-range information missing from standard Illumina sequencing and is then used to assemble phased assemblies *de novo*. 10x Chromium libraries require a minimum of 1.25ng of high molecular weight gDNA at a concentration of 1 ng/ul. gDNA should greater than 50kb in length in order to take full advantage of the technology.

Optical mapping (Bionano)

Bionano technology produces optical maps of nicking/restriction enzyme sites across kilobase-long stretches of DNA molecules, providing a high-throughput tool for ordering and orienting contigs of physical maps and validation of genome assemblies [29]. Bionano optical maps can be compared to *in silico* restriction maps produced from an NGS genome assembly for validation purposes, to improve contiguity by assigning the shorter NGS scaffolds to the longer optical maps, and identifying structural variants. 600 ng of raw gDNA at a concentration of 35-200 ng/µl is typically enough DNA to generate about 120 µl of labelled molecules – enough to provide adequate coverage for analysis of a human-sized genome (3 Gb).

Genome contiguity has an effect on what analyses can be achieved (Table 1), so it is important to appreciate the power and limitations of each sequencing strategy and technology.

Assembly resolution	Paired-end	Paired-end + Long Mate Pair	Bionano	10x Genomics
Gene content	Yes	Yes	No	Yes
Gene order	Yes	Yes	No	Yes
Repeat spanning	No	Yes	Yes	Yes
Structural variants	No	Yes	Yes	Yes
Haplotype resolution (phased genomes)	No	No	No	Yes

Table 1. Information regarding the possible resolution for various *de novo* genome sequencing technologies. When planning a genome assembly project, it is important to understand the strengths and limits of the various sequencing strategies available.

Materials and Methods

Sequencing

Using the same sample of a roadkill European Polecat stored in 100% ethanol, two lanes of PCR-free Illumina HiSeq2500 250bp PE reads (77x coverage), two Illumina LMP libraries of size 5 kb (27x coverage) and 7 kb (9x coverage) and four lanes of 150bp PE 10x Genomics Chromium (totalling 85x coverage) using an Illumina HiSeq2500 were generated (Illumina HiSeq 2500 System, RRID:SCR_016383).

We extracted DNA from four European Polecat samples (all from the VWT collection) and analysed the molecule distribution using an Agilent TapeStation (Supplementary Figure S3). Sample VWT 693 had the highest concentration of the longest molecules where the distribution of molecule lengths peaked at just under 60kb and was used for all further sequencing. For this sample 50% of the molecules were greater than 51kb. The mean molecule length of the remaining 50% of molecules (i.e. those less than 51kb) was 15kb. This was not of good enough quality to generate Bionano data (recommended >150kb). Because the domestic ferret and its polecat ancestor diverged only around 2000 years ago, and fully interbreed we do not expect significant divergence and structural differences between the two species [19, 30-34]. Therefore, the original sample used for the domestic ferret genome assembly [35] was obtained and one chip of Bionano Genomics optical genome maps was generated (Saphyr, RRID:SCR_017992). This was used to create Bionano hybrid-scaffold assemblies for the European Polecat genomes assembled with the previously mentioned short-read data, using the Bionano Solve software [36]. We generated 664 Gb of Bionano data, with an N50 size of 185 kb and a contig coverage of 261x. Of this, 40% of the molecules aligned back to the Bionano *de novo* assembly, leaving an effective coverage of

110x. A more detailed description of the library preparation methods can be found in the Supplementary Methods and the protocols are also available in protocols.io [37].

Assemblies

10 different genome assemblies were generated as summarised in Figure 1, (with additional information in Supplementary Table S1), and detailed as follows:

Assembly A1 (w2rap).

The PCR-free Illumina reads from polecat were assembled using the w2rap-contigger [38]. The w2rap-contigger (w2rap) originated from a fork of the popular DISCOVAR de novo program (Discovar, RRID:SCR_016755) [39] and then a number of improvements were made to reduce memory usage and processing time, enhance parameterization, improve repeat resolution, and increase accuracy and contiguity. It also benefits from requiring less computational resources of other popular assemblers such as ALLPATHS-LG (ALLPATHS-LG , RRID:SCR_010742)[40]. w2rap is predominantly a contig assembler - reads are used to construct an assembly graph which is then traversed to create a contig assembly. A final step involves using the PE information to scaffold contigs not joined during the initial assembly process. Using w2rap, four different assemblies were created using a range of k-mers (k=180, 200, 224, and 240) and simple assembly stats were run to examine contiguity across the assemblies (for all contigs and filtering for contigs > 1kb). From these statistics, the assembly constructed with k=224 was selected as the final assembly.

Assembly A2 (w2rap + lmp).

We analysed the distribution and coverage from the 12 Nextera LMP libraries and selected the 5kb and 7kb libraries due to their tight distribution and higher coverage, when compared to the other ten libraries. Using SSPACE (SSPACE, RRID:SCR_005056)[41] the 5 kb and 7

kb Nextera LMPs were used to scaffold the w2rap assembly from assembly A1. For all SSPACE LMP assemblies the reads were used only for scaffolding and not for contig extension.

Assembly A3 (10x).

The 10x Genomics Chromium library was assembled using the 10x Genomics Supernova software [28], using default parameters. Default parameters automatically cap the number of reads to 1,200M which, after trimming and filtering, resulted in an effective coverage of 52.18x with a mean molecule length of 38.42 kb. Similar to w2rap, Supernova creates an initial contig assembly but then scaffolds using the molecule-specific barcode information in the reads to join contigs known to be from the same molecule [28]. The output style of the resulting assembly was ‘pseudohap’, which creates one haplotype per scaffold at random.

Assembly A4 (10x + lmp).

SSPACE was used with the 5 kb and 7 kb Nextera LMPs to scaffold the 10x assembly generated in assembly A3. As in assembly A2, the LMP reads were used only for scaffolding and not for contig extension.

The Bionano data was assembled *de novo* and then was used to position and orient scaffolds from previous assemblies creating a Bionano hybrid-scaffold as follows:

Assembly A5 (w2rap + bionano). Bionano hybrid-scaffolding with w2rap assembly (Assembly A1).

Assembly A6 (w2rap + lmp + bionano). Bionano hybrid-scaffolding with the w2rap + lmp assembly (Assembly A2)

Assembly A7 (10x + bionano). Bionano hybrid-scaffolding with the 10x assembly (Assembly A3).

Assembly A8 (10x + lmp + bionano). Bionano hybrid-scaffolding with the 10x + lmp assembly (Assembly A4).

Finally, the 30x coverage of 10x Genomics data (from the same data generated for assembly A3, henceforth referred to in the text as '10x-scaffolding') was used to scaffold two assemblies using the scaff10x program from Phusion2 [42], as follows:

Assembly A9 (w2rap + 10x). The w2rap-only assembly (Assembly A1) with 10x-scaffolding.

Assembly A10 (w2rap + lmp + bionano + 10x). The w2rap + lmp + bionano assembly (Assembly A6), with 10x-scaffolding.

Figure 1. Ten different assembly strategies using a variety of different data types: PCR-free Illumina short-read ('PCR-free'), long mate-pair ('LMP'), 10x Genomics Chromium library ('10x'), and Bionano Genomics optical maps ('Bionano'). The blue-boxed assemblies all originate from the same PCR-free w2rap assembly (A1) and the black-boxed assemblies all originate from the same 10x Genomics Supernova assembly (A3). Information in brackets refers to assembly software pipeline and assembly numbers are annotated below each assembly.

Analyses

Genome contiguity

For each genome assembly, a number of assembly statistics, such as contig N50, scaffold N50, the number of scaffolds greater than given lengths and scaffolded genome size were

calculated. To calculate contig N50, any scaffolded-contigs that were joined by 25 or more Ns were broken. The percentage of the genome contained in scaffolds greater than 25 kb (the average length of a vertebrate gene [43]), and the number of scaffolds greater than 39 Mb (the length of the smallest chromosome in a recent chromosome-scale assembly of a closely-related mustelid [44]), were also calculated.

K-mer analysis

The K-mer Analysis Toolkit (KAT) version 2.3.4 [45] was used to examine k-mers across reads and assemblies. KAT enables users to assess levels of errors, bias and contamination at various stages of the assembly process. Using the KAT 'comp' program with a k-mer size of 31, k-mers in the PCR-free Illumina reads were compared with those in the resulting assemblies (omitting the Bionano assemblies as this technology adds negligible sequence content) and for each assembly, the k-mer-spectra was plotted.

Gene content

BUSCO (v3.0.2) was used to search for single-copy orthologs in each assembly (BUSCO , RRID:SCR_015008)[46]. BUSCO reports the number of single-copy orthologs discovered in the input assembly, and categorises them as 'complete', 'single-copy', 'multi-copy' or 'fragmented'. Mammalia_odb9 was used for the 'lineage' parameter in BUSCO and 'human' for the Augustus species parameter.

Repeat content

To examine repeat content and compare how repeats were resolved in each genome assembly RepeatMasker (v 4.0.7 with library dc20170127-rb20170127, RRID:SCR_012954) [47] was used (with default values) to identify repeat families in each assembly, using all *Carnivora*-

specific repeats. As well as identifying repeat sequences, the mean deletion, insertion and divergence for each family was also calculated, as well as the mean values overall. Mean divergence is calculated as ‘mismatches/(matches + mismatches)’ between queries and matches for all repeats.

Assembly errors and misassemblies

REAPR (REAPR, RRID:SCR_017625)[48] was used to evaluate the accuracy of each genome assembly by separately mapping PCR-free PE and LMP reads back to each assembly. The fragment coverage distribution (FCD) error for each assembly was calculated. FCD is the fragment depth from only the reads that are mapped to a given base of a fragment. The FCD error is the difference between the theoretical and observed FCD and is used to identify assembly errors in the regions containing a run of high FCD errors. Mapping information such as the FCD and insert size distribution is analysed to locate misassemblies as well as more local per-base accuracies. The ‘smalt map’ option in REAPR was used, which uses SMALT (SMALT, RRID:SCR_005498)[49] to align the PCR-free PE and LMP reads back to each assembly utilising the option to map PE reads independently. This ensures that read pairs are not artificially forced to map as proper pairs within a given insert size. REAPR was then used to identify perfectly and uniquely mapped reads in the PE PCR-free alignment, to accurately call error-free bases in the assembly and further used the LMP reads to identify features consistent with misassemblies. Error-free bases have at least 5X perfect and unique coverage of paired end reads. REAPR summary scores were calculated for each assembly by multiplying the number of error-free bases with the square of the REAPR broken scaffold N50 length, and then dividing by the original scaffold N50, i.e. ‘No. error-free bases * (broken $N50^2$/assembly N50)’. This test was first used to evaluate genome assemblies in the Assemblathon series [43] and rewards local accuracy, overall contiguity

and correct scaffolding of an assembly. In order to independently asses the performance of each datatype for scaffolding the number of REAPR breaks were compared between the w2rap-only assembly (A1) and that assembly scaffolded with one datatype, namely LMP (A2), Bionano (A5) and 10x (A9). The same analyses were also performed using the 10x-only assembly (A3).

Value-for-money

Cost is a huge factor in research and ultimately, impacts on decisions made regarding the technologies used. A metric was created to reflect 'value-for-money' by estimating the cost of each assembly and the N50 achieved. This metric is provided as N50/$1K and calculated for contig N50, scaffold N50 and the REAPR broken scaffold N50.

Ranking assemblies

Each assembly was given a rank-score according to its position in each of the seven metrics. The top-placed assembly that performed best in a given metric, was given a rank-score of 10, the second-placed assembly was given a rank-score of 9, and so on, down to the bottom-placed assembly which was given a rank-score of 1.

Assemblies were ranked for the following metrics:

1. Scaffold N50
2. REAPR broken scaffold N50
3. Contig N50
4. Percentage of genome represented by scaffolds >25 kb
5. Single-copy BUSCO orthologs
6. REAPR summary score
7. REAPR broken scaffold N50/$1K

Z-scores

Z-scores were used to combine scores from datasets with different means, ranges, and standard deviations and have the benefit of rewarding/penalising those assemblies with exceptionally high/low scores in any one metric. The influence of each of the seven metrics was tested by removing each metric in turn and recalculating the z-score for each assembly. These recalculations were then used to produce error bars for the final z-score figure, by providing the minimum and maximum z-score that might have occurred if any combination of six metrics was used.

Results

Assembly contiguity and connectivity

Assembly Statistics

After assembling the 10 genomes as described in Figure 1, a number of metrics were calculated for each assembly to examine contiguity and connectivity, measured by the lengths and distribution of the scaffolds within each assembly (Table 2). The mean assembly size for all genomes was 2.52Gb, slightly larger than the 2.41Gb assembly of the domestic ferret [35]. 10x-based assemblies erred on having smaller genome assembly sizes (2.46 – 2.50Gb) with the larger assemblies (2.47 – 2.66Gb) being from the PCR-free Illumina-based assemblies.

**No.**	**Assembly**	**No. scaffolds >100 kb**	**No. scaffolds >1 Mb**	**No. scaffolds >39 Mb**	**% genome >= 25kb**	**Longest scaffold (Mb)**	**Contig N50 (kb)**	**Scaffold N50 (Mb)**	**Assembly Size (Gb)**
A1	w2rap	6,290 (10.7%)	176 (0.3%)	0	94.9	2.52	182.93	0.30	2.47
A2	w2rap + lmp	1,680 (4.9%)	682 (2.0%)	0	94.8	15.65	271.16	2.62	2.60

A3	10x	1,023 (3.9%)	501 (1.9%)	0	93.3	32.15	207.98	5.26	2.46
A4	10x + lmp	669 (4.2%)	346 (2.2%)	3	94.7	58.16	210.72	10.33	2.50
A5	w2rap + bionano	4,361 (7.7)	626 (1.1%)	0	93.8	6.89	182.93	0.85	2.66
A6	w2rap + lmp + bionano	990 (3.0%)	468 (1.4%)	0	94.8	34.30	271.16	5.73	2.60
A7	10x + bionano	604 (2.3%)	336 (1.3%)	3	97.5	46.79	207.98	10.84	2.48
A8	10x + lmp + bionano	409 (2.6%)	218 (1.4%)	9	97.6	104.38	210.72	21.01	2.50
A9	w2rap + 10x	1,097 (2.4%)	467 (1%)	0	97.6	35.44	182.93	5.58	2.47
A10	w2rap + lmp + bionano + 10x	447 (1.4%)	235 (0.7%)	6	97.5	65.13	271.16	14.05	2.60

Table 2. Genome assembly statistics (for sequences >1kb) for all assemblies. % scores refer to percentage of scaffolds greater than a given threshold. 39Mb is size of smallest chromosome in a recent chromosome-scale assembly of a closely-related mustelid and hence an indication of the number of chromosome-sized scaffolds. A more thorough list of genome statistics can be found in Supplementary Data Table S2.

Contig N50 for the assemblies varied between 183 kb to 271 kb. Scaffold N50 for the assemblies varied between 300 kb to 21 Mb. The increase from contig N50 to scaffold N50 varied greatly (Figure 2). The addition of LMP data to an initial short-read assembly had a varying effect. On the relatively fragmented w2rap assembly (A1), the addition of LMP reads lead to an almost 9-fold increase of the scaffold N50 but adding LMPs to the more contiguous 10x assembly (A3) resulted in a 2-fold increase. This is not unexpected as the N90 value for the 10x assembly (800kb) is 20 times greater than that of the w2rap assembly (40kb), hence the chance of mate pairs spanning the same contig and not adding to the contiguity of the assembly is much higher in the already contiguous 10x assembly. The addition of Bionano data to assemblies leads to a similar scaffold N50 increases across all assemblies, namely between a 2 and 2.8-fold increase. Finally, 10x-scaffolding data was added to scaffold assembly A1 (w2rap) and assembly A6 (w2rap + lmp + bionano). As might be expected, the effect of 10x-scaffolding data on less contiguous genomes was greater than that on more contiguous genomes. There was an 18.6-fold increase in N50 between assembly A1 (w2rap) and assembly A9 (w2rap + 10x), whereas the increase in N50 between assembly A6 (w2rap + lmp + bionano) and assembly A10 (w2rap + lmp + bionano + 10x) was less contrasting at 2.5-fold.

Generally speaking, assemblies created with one or two data types, where one of the data types was Illumina short reads, showed the smallest increase from contig N50 to scaffold N50 (Figure 2).

Figure 2. Log-scale lengths of contig N50 (blue) and scaffold N50 (red) of all ten assemblies, sorted (left to right) by scaffold N50.

Assembly errors and misassemblies

REAPR was used to assess the accuracy of the polecat genome assemblies by looking at low-quality regions, breakpoints (Table 3), and summary scores. (Figure 3). The percentage of error-free bases for each assembly varied between 76.05% to 85.9%. All the w2rap-based assemblies were on the low end of the scale (76.05% - 81.09%), whilst 10x-based assemblies were on the high end (84.65 % - 85.9%). Conversely, there was a trend for w2rap-based assemblies to be less affected by misassemblies (excluding those with 10x-scaffolding). Their REAPR broken N50 size reduced between 2% - 64%, whilst 10x-based assemblies reduced in N50 size between 68% - 91%. A similar pattern is seen with the number of FCD errors, where all w2rap-based assemblies (bar A10, with 10x-scaffolding) have less than 8214 FCD errors and all 10x-based assemblies have 9095 errors or more.

No.	Assembly name	% error-free	Original N50 (Mb)	REAPR broken N50 (Mb)	% reduction	FCD errors
A1	w2rap	80.83	0.3	0.29	2	6,065
A2	w2rap + lmp	79.10	2.61	1.13	57	8,213
A3	10x	85.90	5.26	1.69	68	11,379
A4	10x + lmp	85.35	10.33	1.86	82	9,095
A5	w2rap + bionano	76.05	0.85	0.52	38	4,523
A6	w2rap + lmp + bionano	78.38	5.73	2.06	64	7,392
A7	10x + bionano	84.65	10.84	2.0	82	13,068

A8	10x + lmp + bionano	84.75	21	1.86	91	11,531
A9	w2rap + 10x	81.09	5.58	0.57	90	7,601
A10	w2rap + lmp + bionano + 10x	77.80	14.05	1.75	88	9,488

Table 3. REAPR statistics showing the percentage of error-free bases in the assembly, N50s before and after breaking at breakpoints, the percentage decrease in scaffold N50 after breaking and the fragment coverage distribution errors (FCD errors) including errors across gaps.

Figure 3. REAPR summary scores for each polecat assembly. REAPR summary scores were calculated for each assembly by multiplying the number of error-free bases with the square of the REAPR broken scaffold N50 length, and then dividing by the original scaffold N50.

Finally, the performance of each technology was independently assessed for scaffolding by comparing the number of REAPR breaks between the w2rap assembly (A1) and those scaffolded with only one datatype (LMP, Bionano, and 10x-scaffolding) (Table 4). After accounting for the 2,756 breaks introduced by REAPR in the w2rap-only assembly (A1), it was found that Bionano (assembly A5) clearly performed best, containing only 729 more breaks than the original assembly (A1). Conversely, LMP (6843 more breaks) and 10x-scaffolding (7,353 more breaks) datatypes had at least 9 times more breaks introduced by REAPR than Bionano. A comparison was made between the number of breaks (5,252) in the

10x assembly (A3) to the 10x + lmp assembly (A4) and the 10x + bionano assembly (A7) (Table 5). A similar pattern as above was found, with the LMP assembly having 2,785 more breaks than the 10x assembly but with the Bionano assembly having only 61 more breaks, again demonstrating the accuracy of Bionano for scaffolding.

No.	Assembly name	Assembled sequences	No. seqs after breaking	REAPR breaks
A1	w2rap	929,245	932,001	2,756
A2	w2rap + lmp	887,887	897,486	9,599 (6,843)
A5	w2rap + bionano	927,316	930,801	3,485 (729)
A9	w2rap + 10x	916,014	926,123	10,109 (7,353)

Table 4. Comparison of the number of breaks introduced by REAPR for each of the technologies used to scaffold the w2rap-only assembly (A1). The number of breaks in brackets represent the number of breaks after accounting for the 2,756 breaks introduce into the comparison assembly (A1).

No.	Assembly name	Assembled sequences	No. seqs after breaking	REAPR breaks
A3	10x	26,253	31,505	5,252
A4	10x + lmp	16,018	24,055	8,037 (2,785)
A7	10x + bionano	25,834	31,147	5,313 (61)

Table 5. Comparison of the number of breaks introduced by REAPR for each of the technologies used to scaffold the 10x assembly (A3). The number of breaks in brackets represent the number of breaks after accounting for the 5,252 breaks introduced into the comparison assembly (A3).

Assembly Completeness

K-mer content

'KAT comp' [44] was used to compare k-mers in the Illumina PCR-free reads with k-mers in the non-Bionano assemblies (A1 – A4 and A9). 'KAT plot' was then used to visualise the output (Figure 4 and Supplementary Figure S1). The plots all show a similar distribution of k-mers. The black distribution at the start of the x-axis represents sequencing errors in reads and its increased width represents an increased number of errors in the reads. K-mers in these reads have not been incorporated into the final assembly. The extension of the black line along the x-axis (up to a k-mer-multiplicity of 40 on the x-axis) represents collapsed haplotypes, where k-mers from one side of a bubble in the assembly graph have been removed to construct a linear path through the graph. Any extension of the black line along the x-axis into the main red distribution (>40 k-mer-multiplicity) represents a small number of high-copy k-mers in the reads missing from the assembly. The red area in all graphs represent a normal distribution of k-mers found in the reads and occurring once in the assembly. The absence of any further colours, representing k-mers appearing once in the reads but multiple times in the assembly, reflects the presence of only unique content throughout the assembly, with k-mers in the reads occurring no more than once in the assembly.

Despite all of the assemblies being compared to the PCR-free Illumina short reads, virtually the same distribution of k-mers between the reads and assemblies was observed, showing an almost-identical distribution of k-mers from all the different read sequences and their resulting assemblies. The KAT-plots involving 10x assemblies (Supplementary Figure S1, C and D) are also characterised by some high-copy read k-mers missing from the assemblies. This suggests that the minimum size of contigs included in the final assembly (1kb) may be too high. This may also explain the slightly smaller assembly sizes obtained from the 10x-based assemblies when compared to the w2rap-based assemblies (Table 2).

Figure 4. KAT k-mer plots comparing k-mer content of Illumina PCR-free reads with w2rap assembly (A1). The black area of the graphs represents the distribution of k-mers present in the reads but not in the assembly and the red area represents the distribution of k-mers present in the reads and once in the assembly.

Gene content

BUSCO was used to look at single-copy orthologs in the assemblies (Figure 5) and examine the number of single-copy, duplicated, fragmented and missing orthologs. The number of complete and single-copy orthologs reconstructed varied from 3,748 (91%) in Assembly A1 and 3,885 (95%) in assembly A10. Of the 4104 mammalian orthologs examined 3603 (88%) were found in single copies across every assembly, 65 were missing, 30 were fragmented, and 21 duplicated across all assemblies. The w2rap assembly (A1) had the highest number of missing orthologs (117) and fragmented orthologs (198), probably due to the fragmented nature of the assembly. Adding scaffold datasets improved ortholog reconstruction in all but

one case, where we found that adding only Bionano (A7) or only LMP (A4) data to the 10x assembly (A3) fragmented a few orthologs (6 and 7 respectively) although adding Bionano data to the 10x + lmp assembly (A4, resulting in assembly A8) increased the number of single-copy orthologs by connecting 13 fragmented ones. Generally, the addition of LMP data had the least beneficial effect on ortholog reconstruction, followed by Bionano, and then 10x-scaffolding. Indeed, the lowest ranking assembly (A1) jumped to the second-highest-ranking assembly merely by the additions of 10x-scaffolding data, which reduced the number of fragmented orthologs from 198 to 94.

Figure 5. Number of single-copy (blue), duplicated (orange), fragmented (grey) and missing (yellow) orthologs from BUSCO. In order to visualise the number of duplicated, fragmented and missing orthologs, the first 3,500 single-copy orthologs present in each assembly are truncated.

Repeats

RepeatMasker was used to look at *Carnivora*-specific repeat content in the assemblies. Long Interspersed Nuclear Elements (LINEs) and Short Interspersed Nuclear Elements (SINEs) were by far the most common classes of repeats and these are concentrated on here. A very similar picture was found between all datasets. The percentage of the genome assemblies that were masked for repeats varied between 35.82% - 39.49%, with SINEs varying between 8.4% - 9.81%. The w2rap-based assemblies were on the lower-end of both of these scales with the 10x-based assemblies on the higher-end.

A slightly different pattern was found when examining LINEs, the composition of which varied between 19.2% and 20.73%. In these repeats the w2rap-based assemblies clustered at the lower end of the scale, with the exception of assembly A1 (w2rap) and assembly A9 (w2rap + 10x), which grouped with the 10x assemblies at the higher end of the scale.

Mean divergence between each assembly and all repeat families was also calculated. It was found that the divergence between assemblies was small (24.52 – 24.60), with no defined grouping of the assemblies by datatype. This suggests an overall similar ability of each datatype to accurately reconstruct repeat sequences (Table 8)

No.	Assembly name	% masked	% SINEs	% LINEs	mean divergence
A1	w2rap	38.31	8.99	20.53	24.52
A2	w2rap-lmp	37.24	8.75	19.95	24.56
A3	10x	39.49	9.81	20.73	24.53
A4	10x-lmp	39.1	9.71	20.52	24.54
A5	w2rap-bionano	35.82	8.40	19.20	24.52
A6	w2rap-lmp-bionano	36.79	8.64	19.71	24.52
A7	10x-bionano	39.11	9.72	20.53	24.60
A8	10x-lmp-bionano	38.89	9.66	20.41	24.58
A9	w2rap 10x	38.32	8.99	20.54	24.55
A10	w2rap_lmp_bionano_10x	36.79	8.64	19.70	24.53

Table 8. Repeat content of assemblies. % masked refers to the amount of the genome masked for all repeats, % SINEs and % LINEs reflect the percentage of the genome found to contain each of these classes, and mean divergence is calculated as 'mismatches/(matches + mismatches)' between queries and matches for all repeats.

Value-for-money

The N50/$1K metric (see Methods) was calculated in order to provide a metric for value-for-money when considering the choice of technology and the return on money spent (Figure 6). For contig N50/$1K, the w2rap-based assemblies provide by far the best value-for-money, with the exception of those with 10x-scaffolding. Value-for-money decreases as more data is added to the w2rap assemblies. So, for contig assemblies a basic PCR-free Illumina short-read assembly provides the best value-for-money.

However, when looking at scaffold N50/$1K, the trend changes. Five of the six lowest-scoring assemblies constitute w2rap-based assemblies, generated with between one and three datatypes. The 10x-based assemblies show better performance when looking at scaffold N50/$1K, with three of the four highest-scoring assemblies being 10x-based. The difference in scaffold N50 between the w2rap-based and 10x-based assemblies might be expected as the short-read Illumina data does not contain the additional molecule specific linked-read information present in 10x data. Another trend is that adding more scaffolding data to a 'base' assembly (A1 and A3) increased the scaffold N50/$1K. Hence, adding more data to increase scaffold contiguity provides value for money, although one must judge if the amount of increase justifies the extra cost.

Figure 6. N50/$1K, providing an estimate to the cost of contig (orange), scaffold (blue) and REAPR broken scaffold (yellow) contiguity for each genome assembly. Assemblies are ranked in order of scaffold N50/$1K.

Ranking assemblies

Assemblies were ranked on a number of key metrics (see Methods), allocated a final rank-score (Supplementary Figure S2) and z-scores were calculated for each assembly (Figure 7). The order of ranking in the rank-scoring method is similar to the z-score ranking, although when the z-scores are calculated the 10x assembly (A3) and w2rap + lmp + bionano + 10x assembly (A10) both rank two places higher, with the 10x + lmp + bionano assembly (A8) ranking a place lower. The z-scores provide a better assessment of the performance of each assembly across all the metrics and not just their position in the final ranking. The general trend was that the more data included, the higher the assembly ranked, although this was not always the case. For example, the second-highest ranked assembly was A10, the only assembly with four different data types (w2rap + lmp + bionano + 10x), but the highest placed assembly was assembly A6 (assembly A10, but without the final 10x scaffolding data). Also, the 10x-only assembly (A3) ranked one place higher than the 10x + lmp assembly (A4).

Figure 7. Cumulative z-scores of assemblies (solid black circles) with error bars (blue). Error bars represent the min and max cumulative z-score after removing each metric in turn and recalculating the z-score for each assembly. Wide error bars show assemblies that are

strongly affected by a given metric. For example, the 10x + lmp + bionano assembly (A8) has a long lower-boundary error-bar as it has an exceptionally high scaffold N50 z-score (double that of the next nearest ranking assembly) and hence omitting this metric results in the assembly scoring much lower.

Discussion

Although chromosome-scale assemblies are now achievable, it is often not possible or necessary to assemble the genomes of non-model organisms to such precision. A number of difficulties are faced when sequencing and assembling non-model organisms. Genome size (and as a consequence, sequencing depth), chromosome number, sequence composition and GC content are often unknown or inaccurate, the species can be highly heterozygous, and samples are often degraded. We address some of these factors and identify which sequencing and assembly strategies are required to answer various biological questions. PCR-free Illumina short-read, 10x Genomics linked-read, long mate paired read, and Bionano optical maps were generated from a roadkill European Polecat to create ten different genome assemblies, using different combinations of the data. The assemblies were assessed using a range of tools and ranked using seven key metrics. We find that although some genomes assemble to high contiguity, this is often at the expense of accuracy and it is often not necessary to spend additional funds on increasing contiguity to answer biological questions.

Assembly contiguity and connectivity

As a general rule, adding more data to an assembly increases the contiguity (scaffold N50). This was observed in the assemblies here, with each assembly having a higher scaffold N50 than any 'parent' assembly before it. The linked reads from 10x Genomics data constantly outperform the equivalent PCR-free short-read-based assemblies, with the barcoded linked

reads acting as an additional scaffolding dataset. The assemblies with the best contig N50 were those based on w2rap + lmp (namely, A2, A6 and A10). For scaffold N50 and percentage of the genome represented by scaffolds >25Kb, 10x + lmp + bionano (A8) provided by far the best contiguity. When REAPR breaks are taken into consideration, the w2rap + lmp + bionano assembly (A6) provides the best scaffold N50, although assembly A8, with the best initial contig N50, is still ranked third. It should be noted that Bionano and 10x (for scaffolding) added no sequence data to the assemblies and hence do not extend the contig lengths or have not connected contigs together without the need to add 'N's. Those assemblies scaffolded with LMPs however do increase in contig N50, reflecting previously unconnected contigs being joined without Ns.

An increase in REAPR summary scores was seen when LMP and Bionano data are added to PCR-free short read assemblies, but a decrease in summary scores when 10x-scaffolding reads are included. For 10x-based assemblies, the addition of extra data leads to a reduction in summary scores. Additionally, 10x-based assemblies tended to have more FCD errors and the breaking of assemblies at these errors affected 10x-based assemblies to a greater degree than w2rap-based assemblies. Finally, the number of breaks created by REAPR for each scaffolding technology showed that Bionano-scaffolded genomes had significantly fewer breaks than both LMPs and 10x-scaffolding. The addition of 10x-scaffolding data led to an overall reduction in summary scores suggesting that although 10x-scaffolded assemblies provide a good increase in scaffold N50, much of this increase is through misassemblies.

The increase in misassemblies with the addition of extra data is understandable. An initial, one-technology *de novo* assembly will have all the 'easy joins' put together and most of those will be correct. When a new datatype is added, it will have the 'difficult joins' to put

together, making it very likely that a significant number of these will be incorrect. Bionano performs best at connecting these ‘difficult joins’.

Assembly completeness

Gene content usually increased after adding scaffolding data, with the exception of the 10x assembly. Here, adding only LMP data or only Bionano data fragmented a few orthologs, but incorporating both technologies led to an increase in ortholog reconstruction. Other than this it is clear that 10x data performs exceptionally well in the gene space, both for de novo sequencing and for scaffolding. Bionano also performs well, with LMP data having the smallest impact.

There was a small amount of difference in repeat content between assemblies. The tendency of 10x assemblies to have a slightly higher percentage of the genome assembled as repeats probably reflects the ability of this technology to better resolve repeats than the standard short-read assemblies which collapse a large proportion of the repeats. Those repeats that were resolved in assemblies all showed a very similar divergence, regardless of the data types used.

Value-for-money

For contig assembly, a basic PCR-free short read assembly provides the best value-for-money (A1). Adding more data does not increase the contig N50 enough to warrant the extra expense. For scaffold assembly, the story is very different. The 10x + lmp + bionano (A8) offers the best value for money. The more data added to an initial assembly, the higher the scaffold N50/$1K. When REAPR broken assemblies are taken into consideration, the 10x-based and LMP-scaffolded assemblies provide the best value, with the w2rap + lmp +

bionano assembly (A6) being ranked top (Figure 6). Another feature when considering REAPR broken assemblies, is the poor performance of 10x-scaffolding (A9 and A10). Compared to an Illumina PCR-free library, 10x Chromium libraries are expensive to produce due to higher cost of the library preparation and the additional hands-on time required associated with the protocol. This increased cost for the 10x Genomics scaffolding data and the high misassembly rate when used as a scaffolding technology, means that it scores low in this metric. Nextera LMP libraries are even more expensive to produce than 10x libraries and take a similar amount of preparation time, but are less susceptible to misassembly and score higher in this metric.

In summary, when looking at genome contiguity, if contigs are all that are required from a genome assembly, then PCR-free short-read assemblies with no additional datatypes provide the best value-for-money. If accurate scaffolds are more important, 10x data, often augmented with LMPs or Bionano provide good value-for-money, with Bionano misassembling significantly fewer scaffolds than LMPs.

Ranking assemblies

As expected, the general trend was that the more data included, the higher the assembly ranked, with the addition of Bionano or 10x data providing the most powerful scaffolding technique.

Application to non-model mammals.

Although the sample quality used for non-model organisms is often sub-standard, sequencing technologies and software are still successful in assembling these samples into highly contiguous genomes. As a general rule, adding more data to an assembly increases the

contiguity (scaffold N50), but the additional expense of incorporating additional data to increase contiguity is not always necessary.

For population genetics approaches, SNP-calling and large multi-species comparisons, basic short-read assemblies such as w2rap (A1) or 10x (A3) provides enough accuracy and contiguity to achieve interpretable results. 10x assemblies also have the added advantage of haplotype resolution (phased genomes). Where structural variation, long repeat content, gene order, or gene clusters are of importance an additional scaffolding dataset is often necessary to obtain the required precision for these analyses (A2, A4, A5, A7, and A9), with 10x-scaffolding or Bionano being the better data to incorporate if working in the gene space. Examples where this might be important is when dealing with gene clusters of similar genes, such as immune-related gene clusters (e.g. MHC, Interleukin, toll-like receptors, etc.). When looking at more long-range features, such as genome synteny, Bionano provides additional contiguity. Bionano though, is dependent on high-quality HMW DNA which might not be available for many organisms and appears to be the first datatype to suffer from sample degradation. This was apparent from our polecat sample where the distribution of molecule lengths peaked at less than 60kb, and of which the smallest 50% of material had a mean molecule length was 15kb (Supplementary Figure S3).

Experimental design

Assemblies with both short contig lengths and a high number of misassemblies, can sometimes be found in very heterozygous species. Knowing the distribution of molecule lengths from a sample will provide information about the limitations of which sequencing technologies can be successfully supplied. Researchers can then design their assembly and analysis pipeline to accommodate the limitations of the sample. For example, if the molecule

lengths are only in the region of 1kb, then PCR-free Illumina paired-end sequencing is the only viable option. Longer molecules, between 10 – 40 kb allow the preparation of LMP libraries and between 20 – 100kb permit the inclusion of 10x Genomics data. Beyond that (100kb+), Bionano optical maps may also be included. Low coverage 10x Genomics sequencing has recently been shown to produce a high-quality, cost-effective *de novo* assembly in a non-model mammal [50]. Using 25x coverage, a *de novo* assembly of the African wild dog produced a reference genome with contig and scaffold N50s of 50kb and 15.3Mb respectively, providing another avenue of assembly approach.
Recently, Hi-C sequencing has also been used to good effect to scaffold genomes of a number of different organisms [51-54]. The technique is based on cross-linking DNA, then digesting the DNA with restriction enzymes. The DNA fragment ends are then re-ligated, which will contain two fragments of DNA that were far apart in the genome but still maintaining some degree of physical proximity (e.g. on the same molecule). By sequencing the ends of these fragments, paired-end reads can be mapped to *de novo* genome assemblies and used to scaffold and order contigs, creating chromosomes-scale assemblies [55, 56]. Although the Hi-C protocol does not specify a minimum molecule length (the protocol is carried out in cells or tissue), it relies on fresh DNA long enough to form distant cross-linked fragments.

Adding long-read data, such as low-coverage PacBio or Nanopore data, will often be the only solution to overcoming complexities such as high heterozygosity or long repeats. Unfortunately, long-read data relies on high molecular weight DNA with long molecules, but as described previously, DNA samples from non-model organisms are often of low-quality and the application of these technologies may not be suitable. The quality of sample should reflect the experimental design and assembly pipeline. Development in new DNA extraction

(e.g. Nanobind Magnetic Disks [57]) and sequencing technologies may provide access to low quantity and quality of DNA, which may be a potential solution to overcome the sample extraction issues.

As mentioned, with longer molecules, using long-read technologies such as PacBio and Nanopore becomes a possibility, but these require significantly more DNA (>20ng) to work successfully, as well as being associated with a much higher cost. This overcomes some of the limitations of short-read assemblies, such as characterising structural variation, sequencing through extended repetitive regions, discriminate paralogous genes and detecting disease-associated mutations, although with the drawback of requiring high-coverage due to the lower base accuracy of long read sequencing.

Limitations of this study

In this study a combination of four different technologies have been used to create 10 different genome assemblies. An exhaustive assessment would produce many more different assemblies, so a choice of what was considered a good representation of all practical combinations was used. Additionally, different assembly software (including versions thereof) may produce slightly different results depending on the algorithms used within them. Finally, test metrics can bias results. For example, the inclusion of more cost-related metrics would bias rankings to favour cheaper assemblies, whereas more contiguity-related tests would bias results for assemblies with higher N50s. The choice of metrics was made to encapsulate genome contiguity, accuracy, error, biologically meaningful content, and cost whilst not unduly biasing the results towards any one feature of the assemblies.

Summary

We address how different sequencing and assembly strategies are required to answer various biological questions in non-model mammals. We find that although some genomes assemble to high contiguity, this is often at the expense of accuracy and it is often not necessary to spend additional funds on increasing contiguity to answer biological questions.

Sequencing technologies and assembly software are always progressing with new sequencing chemistry releases providing longer and more accurate read sequences. Also, novel assembly algorithms promise more contiguous and accurate assemblies. Often each algorithm is dependent on the input of specific data types, with some new assembly software providing more contiguous assemblies at the expense of accuracy. It is important to fully assess the performance of an assembly by using a number of different quality assessment approaches as shown in our study, rather than relying on simple statistics such as scaffold N50, which itself can be biased by the exclusion of shorter sequences from the calculations.

Finally, given the accuracy of PCR-free assemblies and the contiguity of the 10x linked-read technology, if a PCR-free linked-read sequencing technology existed, it would provide accurate, contiguous and cheap assemblies.

Data availability

The submission of sequencing data was brokered by the COPO platform (https://copo-project.org), funded by the BBSRC (BB/L024055/1) and supported by CyVerse UK, part of the Earlham Institute National Capability in e-Infrastructure. All datasets supporting the results of this article are available in the ENA repository under umbrella project accession number PRJEB34131. Optical maps, annotations and other results are available from the *GigaScience* GigaDB repository [57], and protocols are available from protocols.io [37].

Competing interests

The authors declare that they have no competing interests.

Funding

This work was strategically funded by the BBSRC Core Strategic Programme Grant BBS/E/T/000PR9817 at the Earlham Institute (EI). High-throughput sequencing and library construction was delivered via the BBSRC National Capability in Genomics (BB/CCG1720/1) by members of the Genomic Pipelines Group. This research was supported in part by the NBI Computing infrastructure for Science (CiS) group through the use of the EI High Performance Computing facilities.

References:

1. Pennisi, E., *New technologies boost genome quality.* Science, 2017. **357**(6346): p. 10-11 DOI: 10.1126/science.357.6346.10.
2. Lewin, H.A., et al., *Earth BioGenome Project: Sequencing life for the future of life.* Proc Natl Acad Sci U S A, 2018. **115**(17): p. 4325-4333 DOI: 10.1073/pnas.1720115115.
3. Keller, I., et al., *Population genomic signatures of divergent adaptation, gene flow and hybrid speciation in the rapid radiation of Lake Victoria cichlid fishes.* Mol Ecol, 2013. **22**(11): p. 2848-63 DOI: 10.1111/mec.12083.
4. Prufer, K., et al., *The bonobo genome compared with the chimpanzee and human genomes.* Nature, 2012. **486**(7404): p. 527-31 DOI: 10.1038/nature11128.
5. Jones, F.C., et al., *The genomic basis of adaptive evolution in threespine sticklebacks.* Nature, 2012. **484**(7392): p. 55-61 DOI: 10.1038/nature10944.
6. Shao, C., et al., *Genome-wide SNP identification for the construction of a high-resolution genetic map of Japanese flounder (Paralichthys olivaceus): applications to QTL mapping of Vibrio anguillarum disease resistance and comparative genomic analysis.* DNA Res, 2015. **22**(2): p. 161-70 DOI: 10.1093/dnares/dsv001.
7. Dodds, P.N. and J.P. Rathjen, *Plant immunity: towards an integrated view of plant-pathogen interactions.* Nat Rev Genet, 2010. **11**(8): p. 539-48 DOI: 10.1038/nrg2812.

8. Shaffer, H.B., et al., *Conservation genetics and genomics of amphibians and reptiles.* Annu Rev Anim Biosci, 2015. **3**: p. 113-38 DOI: 10.1146/annurev-animal-022114-110920.
9. Kohn, M.H., et al., *Genomics and conservation genetics.* Trends Ecol Evol, 2006. **21**(11): p. 629-37 DOI: 10.1016/j.tree.2006.08.001.
10. Attard, C.R.M., et al., *From conservation genetics to conservation genomics: a genome-wide assessment of blue whales (Balaenoptera musculus) in Australian feeding aggregations.* R Soc Open Sci, 2018. **5**(1): p. 170925 DOI: 10.1098/rsos.170925.
11. Allendorf, F.W., P.A. Hohenlohe, and G. Luikart, *Genomics and the future of conservation genetics.* Nat Rev Genet, 2010. **11**(10): p. 697-709 DOI: 10.1038/nrg2844.
12. Murgarella, M., et al., *A First Insight into the Genome of the Filter-Feeder Mussel Mytilus galloprovincialis.* Plos One, 2016. **11**(3).
13. Ekblom, R., et al., *Genome sequencing and conservation genomics in the Scandinavian wolverine population.* Conserv Biol, 2018. **32**(6): p. 1301-1312 DOI: 10.1111/cobi.13157.
14. Zhao, S., et al., *Whole-genome sequencing of giant pandas provides insights into demographic history and local adaptation.* Nat Genet, 2013. **45**(1): p. 67-71 DOI: 10.1038/ng.2494.
15. Locke, D.P., et al., *Comparative and demographic analysis of orang-utan genomes.* Nature, 2011. **469**(7331): p. 529-33 DOI: 10.1038/nature09687.
16. Miller, W., et al., *Polar and brown bear genomes reveal ancient admixture and demographic footprints of past climate change.* Proc Natl Acad Sci U S A, 2012. **109**(36): p. E2382-90 DOI: 10.1073/pnas.1210506109.
17. Der Sarkissian, C., et al., *Evolutionary Genomics and Conservation of the Endangered Przewalski's Horse.* Curr Biol, 2015. **25**(19): p. 2577-83 DOI: 10.1016/j.cub.2015.08.032.
18. Zerbino, D.R. and E. Birney, *Velvet: Algorithms for de novo short read assembly using de Bruijn graphs.* Genome Research, 2008. **18**(5): p. 821-829 DOI: 10.1101/gr.074492.107.
19. Blandford, P.R.S., *Biology of the Polecat Mustela-Putorius - a Literature-Review.* Mammal Review, 1987. **17**(4): p. 155-198 DOI: DOI 10.1111/j.1365-2907.1987.tb00282.x.
20. Croose, E., et al., *A review of the status of the Western polecat Mustela putorius: a neglected and declining species?*, in *Mammalia*. 2018DOI: 10.1515/mammalia-2017-0092.
21. Croose, E., *The Distribution and Status of the Polecat (Mustela putorius) in Britain 2014-2015*. 2016, The Vincent Wildlife Trust.
22. Bentley, D.R., et al., *Accurate whole human genome sequencing using reversible terminator chemistry.* Nature, 2008. **456**(7218): p. 53-9 DOI: 10.1038/nature07517.
23. Kozarewa, I., et al., *Amplification-free Illumina sequencing-library preparation facilitates improved mapping and assembly of (G+C)-biased genomes.* Nat Methods, 2009. **6**(4): p. 291-5 DOI: 10.1038/nmeth.1311.
24. Clavijo, B.J., et al., *An improved assembly and annotation of the allohexaploid wheat genome identifies complete families of agronomic genes and provides genomic evidence for chromosomal translocations.* Genome Res, 2017. **27**(5): p. 885-896 DOI: 10.1101/gr.217117.116.

25. Aird, D., et al., *Analyzing and minimizing PCR amplification bias in Illumina sequencing libraries.* Genome Biol, 2011. **12**(2): p. R18 DOI: 10.1186/gb-2011-12-2-r18.
26. Heavens, D., et al., *A method to simultaneously construct up to 12 differently sized Illumina Nextera long mate pair libraries with reduced DNA input, time, and cost.* Biotechniques, 2015. **59**(1): p. 42-5 DOI: 10.2144/000114310.
27. Leggett, R.M., et al., *NextClip: an analysis and read preparation tool for Nextera Long Mate Pair libraries.* Bioinformatics, 2014. **30**(4): p. 566-8 DOI: 10.1093/bioinformatics/btt702.
28. Weisenfeld, N.I., et al., *Direct determination of diploid genome sequences.* Genome Res, 2017. **27**(5): p. 757-767 DOI: 10.1101/gr.214874.116.
29. Hastie, A.R., et al., *Rapid genome mapping in nanochannel arrays for highly complete and accurate de novo sequence assembly of the complex Aegilops tauschii genome.* PLoS One, 2013. **8**(2): p. e55864 DOI: 10.1371/journal.pone.0055864.
30. Costa, M., et al., *The genetic legacy of the 19th-century decline of the British polecat: evidence for extensive introgression from feral ferrets.* Molecular Ecology, 2013. **22**(20): p. 5130-5147 DOI: 10.1111/mec.12456.
31. Davison, A., et al., *Hybridization and the phylogenetic relationship between polecats and domestic ferrets in Britain.* Biological Conservation, 1999. **87**(2): p. 155-161.
32. Birks, J. and A. Kitchener, *The distribution and status of the polecat Mustela putorius in Britain in the 1990s.* The Vincent Wildlife Trust, London, 1999. **152**.
33. Volobuev, V., *Taxonomic status of ferret based on karyological data.* Zool J, 1974. **53**: p. 1738-1739.
34. Sato, J.J., et al., *Phylogenetic relationships and divergence times among mustelids (Mammalia: Carnivora) based on nucleotide sequences of the nuclear interphotoreceptor retinoid binding protein and mitochondrial cytochrome b genes.* Zoolog Sci, 2003. **20**(2): p. 243-64 DOI: 10.2108/zsj.20.243.
35. Peng, X., et al., *The draft genome sequence of the ferret (Mustela putorius furo) facilitates study of human respiratory disease.* Nat Biotechnol, 2014. **32**(12): p. 1250-5 DOI: 10.1038/nbt.3079.
36. Bionano Genomics, *Bionano Solve*. 2019. https://*bionanogenomics*.com/support-page/*bionano-solve*/
37. Graham J Etherington, Darren Heavens, David Baker, Ashleigh Lister, Rose McNelly, Gonzalo Garcia, Bernardo Clavijo, Iain Macaulay, Wilfried Haerty, Federica Di Palma (2020). Protocols for "Sequencing smart: De novo sequencing and assembly approaches for a non-model mammal". **protocols.io** dx.doi.org/10.17504/protocols.io.bd3ri8m6
38. Clavijo, B.J., et al., *An improved assembly and annotation of the allohexaploid wheat genome identifies complete families of agronomic genes and provides genomic evidence for chromosomal translocations.* bioRxiv, 2016: p. 080796.
38. Love, R.R., et al., *Evaluation of DISCOVAR de novo using a mosquito sample for cost-effective short-read genome assembly.* BMC Genomics, 2016. **17**: p. 187 DOI: 10.1186/s12864-016-2531-7.
39. Gnerre, S., et al., *High-quality draft assemblies of mammalian genomes from massively parallel sequence data.* Proc Natl Acad Sci U S A, 2011. **108**(4): p. 1513-8 DOI: 10.1073/pnas.1017351108.
40. Boetzer, M., et al., *Scaffolding pre-assembled contigs using SSPACE.* Bioinformatics, 2011. **27**(4): p. 578-9 DOI: 10.1093/bioinformatics/btq683.
41. Mullikin, J.C. and Z.M. Ning, *The phusion assembler.* Genome Research, 2003. **13**(1): p. 81-90 DOI: 10.1101/gr.731003.

42. Bradnam, K.R., et al., *Assemblathon 2: evaluating de novo methods of genome assembly in three vertebrate species.* Gigascience, 2013. **2**(1): p. 10 DOI: 10.1186/2047-217X-2-10.
43. Kliver, S., et al. *Chromosome-Level Assembly of the Endangered Black-Footed Ferret (Mustela nigripes) Provides Insights into Male Infertility*. in *Plant and Animal Genome XXVII Conference (January 12-16, 2019)*. PAG.
44. Mapleson, D., et al., *KAT: a K-mer analysis toolkit to quality control NGS datasets and genome assemblies.* Bioinformatics, 2016 DOI: 10.1093/bioinformatics/btw663.
45. Simao, F.A., et al., *BUSCO: assessing genome assembly and annotation completeness with single-copy orthologs.* Bioinformatics, 2015. **31**(19): p. 3210-2 DOI: 10.1093/bioinformatics/btv351.
46. Smit, A., R. Hubley, and P. Green, *RepeatMasker Open-4.0.* 2013-2015.
47. Hunt, M., et al., *REAPR: a universal tool for genome assembly evaluation.* Genome Biol, 2013. **14**(5): p. R47 DOI: 10.1186/gb-2013-14-5-r47.
48. Ponstingl, H., *SMALT*. 2010.
49. Armstrong, E.E., et al., *Cost-effective assembly of the African wild dog (Lycaon pictus) genome using linked reads.* Gigascience, 2019. **8**(2) DOI: 10.1093/gigascience/giy124.
50. Guo, Y., et al., *A chromosomal-level genome assembly for the giant African snail Achatina fulica.* Gigascience, 2019. **8**(10) DOI: 10.1093/gigascience/giz124.
51. Kang, S., et al., *Chromosomal-level assembly of Takifugu obscurus (Abe, 1949) genome using third-generation DNA sequencing and Hi-C analysis.* Mol Ecol Resour, 2019 DOI: 10.1111/1755-0998.13132.
52. Xu, Z. and J.R. Dixon, *Genome reconstruction and haplotype phasing using chromosome conformation capture methodologies.* Brief Funct Genomics, 2019 DOI: 10.1093/bfgp/elz026.
53. Zhou, Y., et al., *Chromosome genome assembly and annotation of the yellowbelly pufferfish with PacBio and Hi-C sequencing data.* Sci Data, 2019. **6**(1): p. 267 DOI: 10.1038/s41597-019-0279-z.
54. Belton, J.M., et al., *Hi-C: a comprehensive technique to capture the conformation of genomes.* Methods, 2012. **58**(3): p. 268-76 DOI: 10.1016/j.ymeth.2012.05.001.
55. Korbel, J.O. and C. Lee, *Genome assembly and haplotyping with Hi-C.* Nat Biotechnol, 2013. **31**(12): p. 1099-101 DOI: 10.1038/nbt.2764.
56. Liu, K., et al., *Nanobind magnetic disksrapid high mw DNA extraction from plant, insect, cell and tissue samples for long-read sequencing using Nanoind Magnetic Disks*, in *Plant and Animal Genome XXVII*. 2019: San Diego.
57 Etherington GJ; Heavens D; Baker D; Lister A; McNelly R; Garcia G; Clavijo B; Macaulay I; Haerty W; Di Palma F (2020): Supporting data for "Sequencing smart: De novo sequencing and assembly approaches for a non-model mammal." GigaScience Database. http://dx.doi.org/10.5524/100731

Figure 1

Click here to access/download;Figure;Figure1.jpg

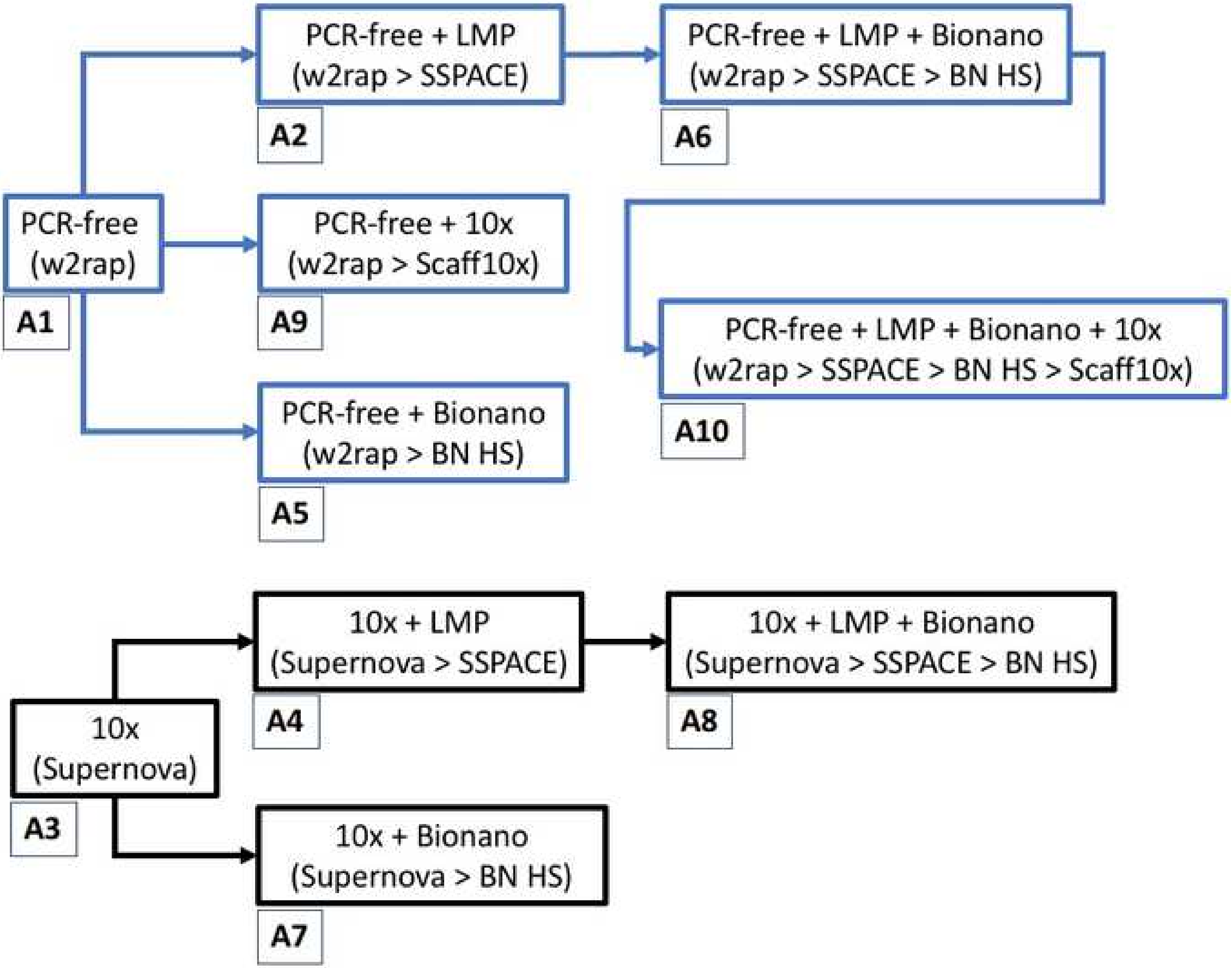

Figure 2

Click here to access/download;Figure;Figure2.png

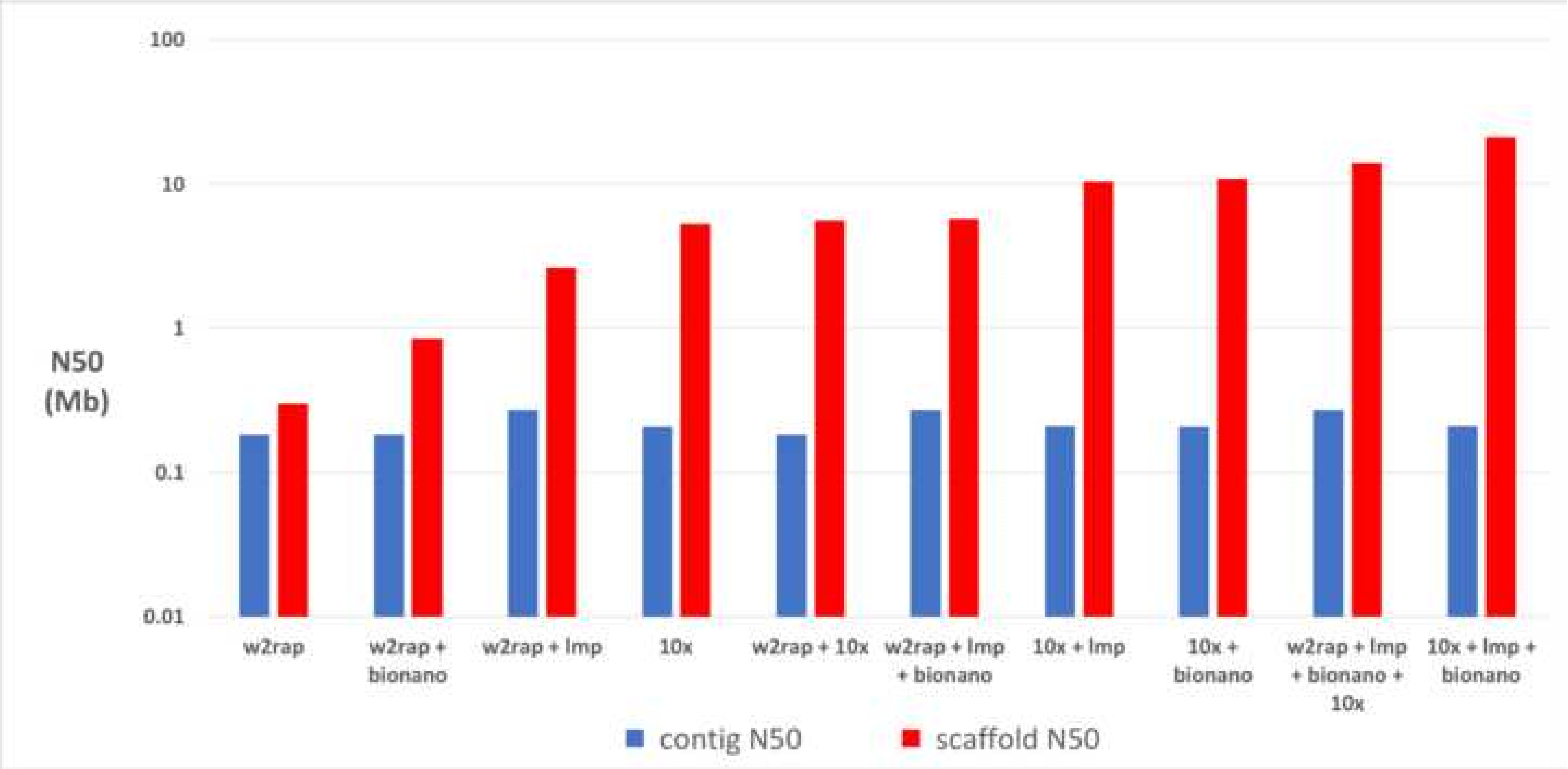

Figure 3

Click here to access/download;Figure;Figure3.png

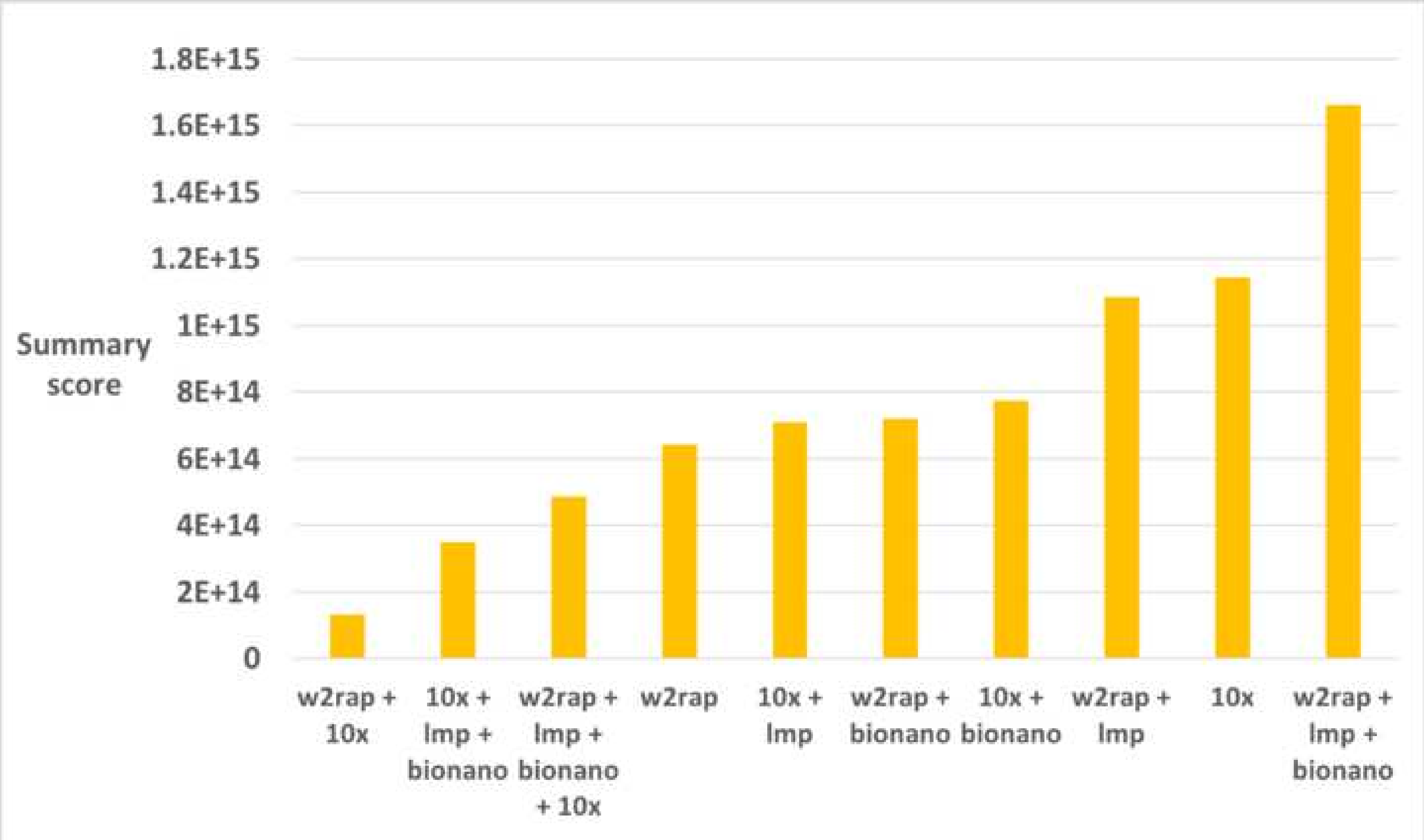

Figure 4

Click here to access/download;Figure;Figure4.png

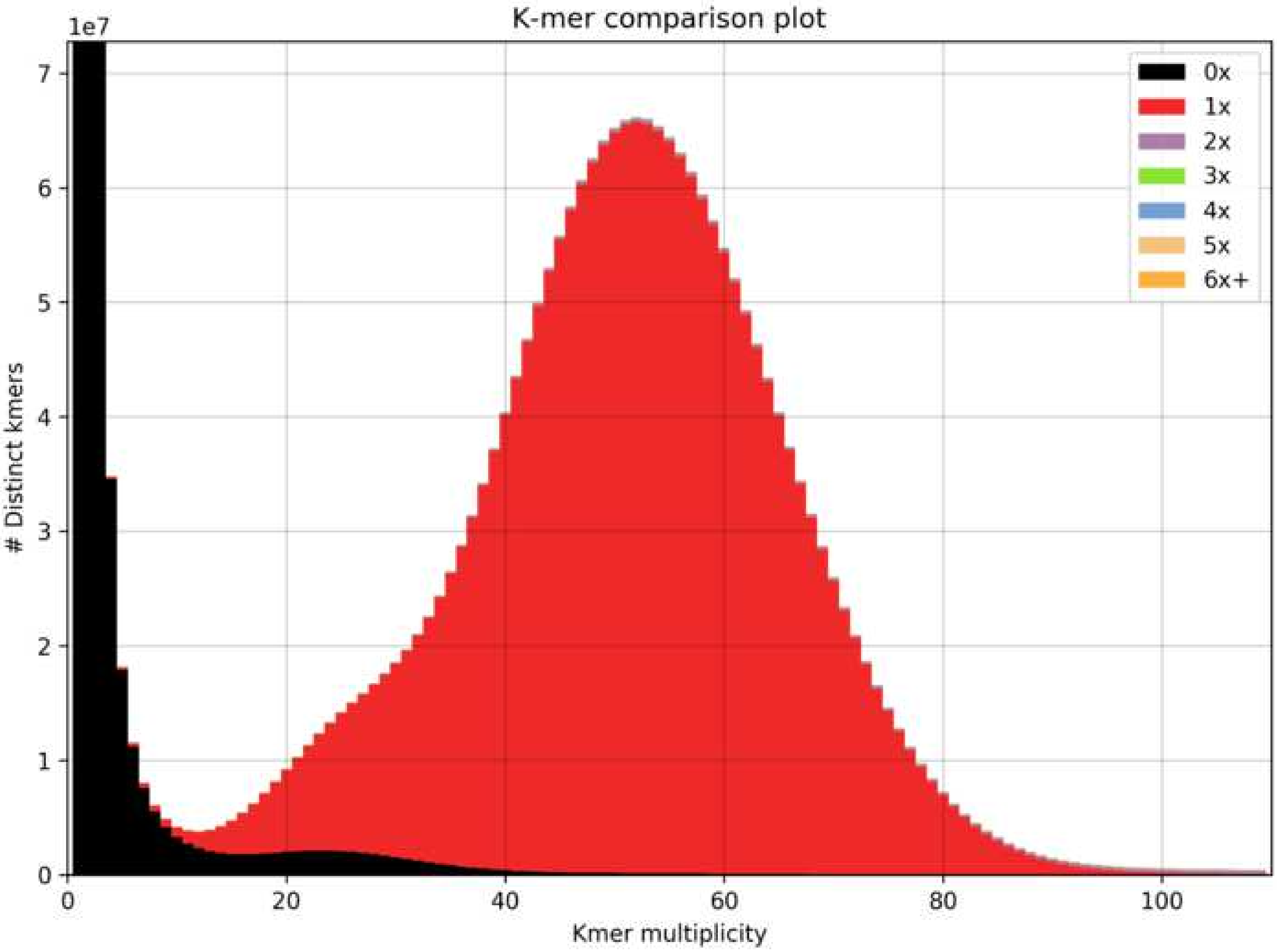

Figure 5

Click here to access/download;Figure;Figure5.png

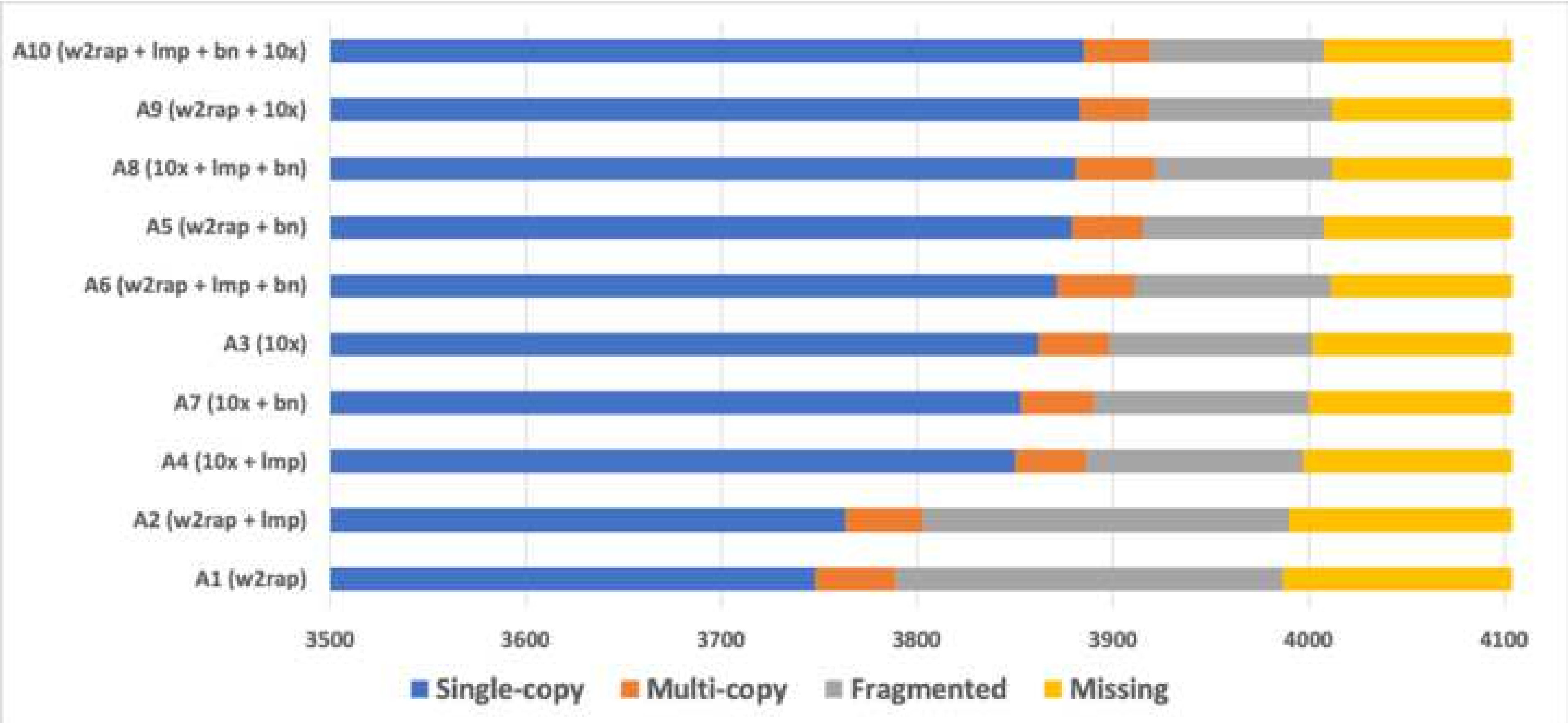

Figure 6

Click here to access/download;Figure;Figure6.png

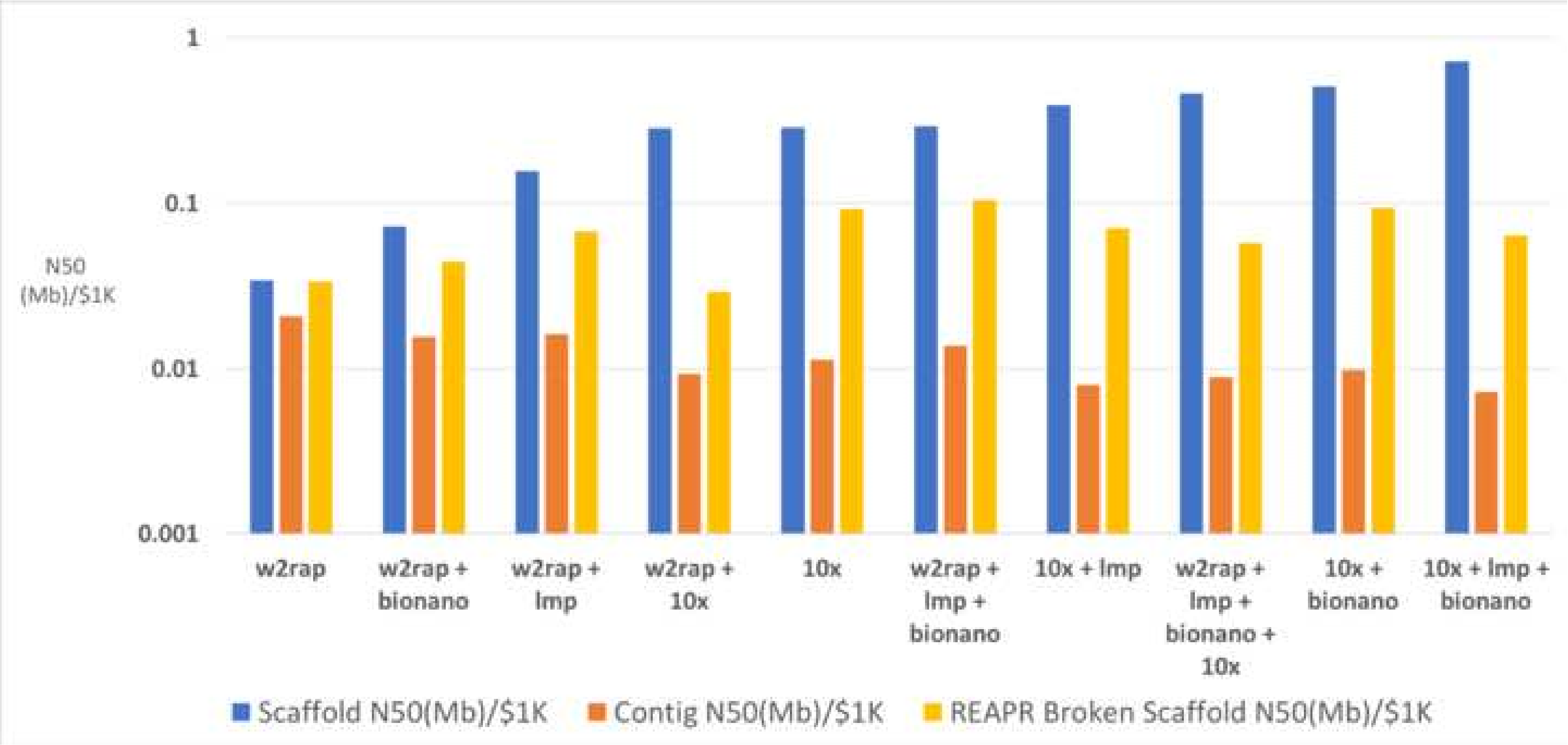

Figure 7

Click here to access/download;Figure;Figure7.png

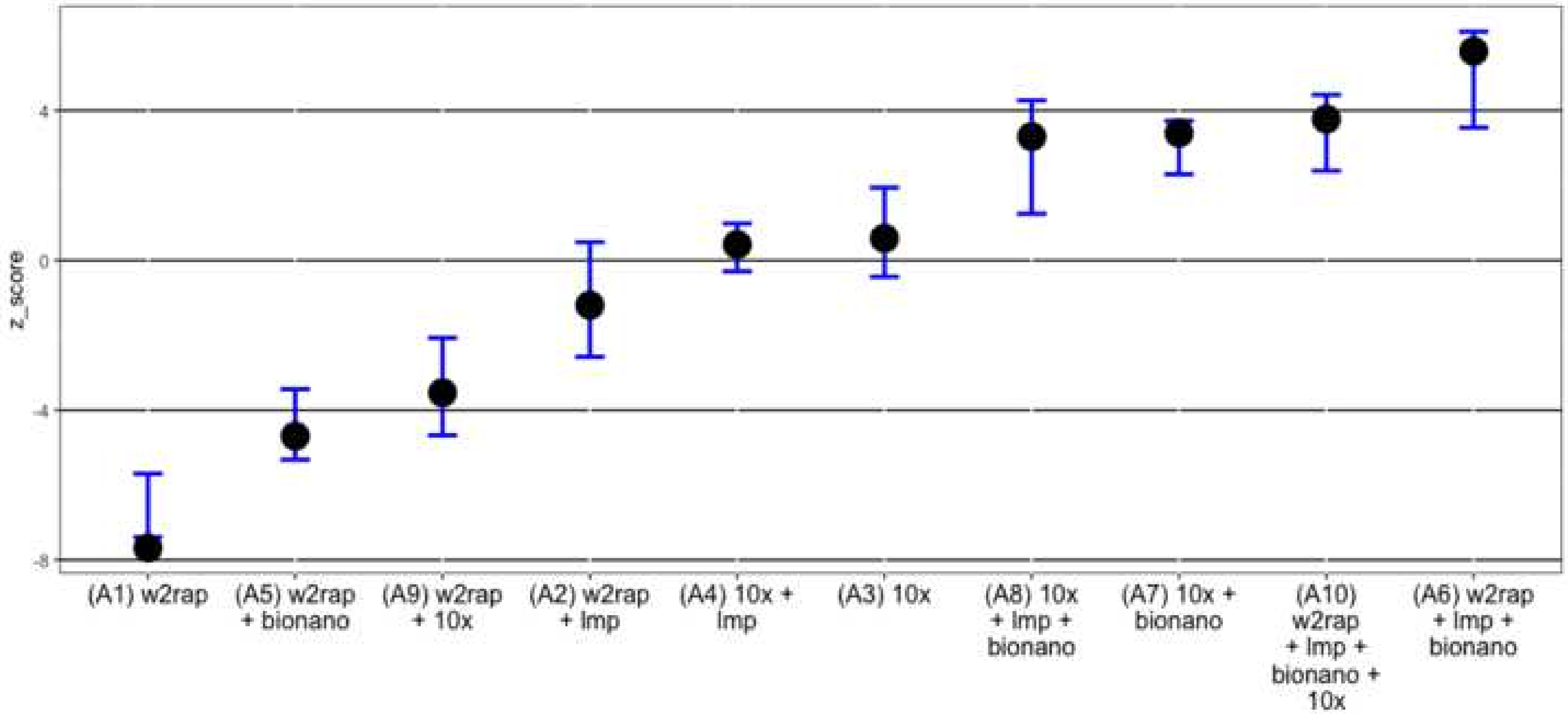

Supplementary table S2

Click here to access/download
Supplementary Material
Supplementary_Data_Table_S2.xlsx

Supplementary data

Click here to access/download
Supplementary Material
Supplementary_Data.docx

Supplementary methods

Click here to access/download
Supplementary Material
Supplementary_Methods.docx

 Click here to access/download;Personal Cover;Response2.docx

Dear GigaScience,
Thank you for your recent communication and for the minor edits suggested by the two reviewers.
Below, we have addressed and responded to each comment in turn.
We wish to once again thank the reviewers for their input and wish them the very best at this most challenging and worrying time.

Reviewer #1:

Comment:
Abstract, Background: "little is known about the correlation between genome sequencing..." I would just write relationship instead of correlation here. Correlation is a bit of a loaded word.
Response:
Correction made.

Comment:
Introduction, top of pg 4: "the cost of generating this amount of data and assemble..." assemble should be "assembling"
Response:
Correction made.

Comment:
Introduction, pg 5, paragraph 2: First sentence reads a bit funny saying that organisms are species from populations, maybe rephrase to: Many samples from non-model organisms originate from wild populations that are highly heterozygous"
Response:
Correction made. Reviewer's suggestion incorporated.

Comment:
Page 6 Sequencing technologies: "Recent machines" to just "Machines"
Response:
Correction made

Comment:
Page 7, Long Mate Pair sequencing: Clarification on this, is 4ug of DNA required to generate all 12 LMP libraries or is this per LMP library?
Response:
4ug of DNA is required for all 12 LMP libraries. We've edited this sentence to read "Nextera LMP sequencing requires a minimum of 4 µg of gDNA for the 12 libraries, at a minimum concentration of 30 ng/µl in 300 µl."

Comment:
Materials and methods, page 9: "Using the same sample of a roadkill European Polecat sample"--delete second "sample", redundant
Response:

Correction made

Comment:
Materials and methods, page 9: You list the coverage for all the libraries except the four lanes of 150bp PE 10x, so maybe just add that in.
Response:
Amended to read “and four lanes of 150bp PE 10x Genomics Chromium (totalling 85x coverage)….”

Comment:
Page 15, Ranking assemblies: The first two sentences of this paragraph can probably be combined, seems repetative?
Response:
We have removed the first sentence and edited the second to read “Each assembly was given a rank-score according to its position in each of the seven metrics.”

Comment:
General comment: It might be useful in your tables and the text to add in ',' to your numbers. For example, 300,334 instead of 300334. I find that with a lot of big numbers it is easier to read this way and quickly get a sense for the magnitude of the difference, i.e. if its 1,000,000 vs 100,000 makes it easier to see that is larger than if you just have 1000000 vs 100000.
Response:
Where appropriate, we have inserted commas for numbers >= 1000. With this in mind, we have also edited Table 3 and changed all the N50 values from bases to Mb (so that it’s a bit less congested with long numbers).

Comment:
Materials and methods, page 9: "We generated 664 Gb of Bionano molecules" --just checking on how this is supposed to be referred to. Do you generate "Bionano molecules" or "Bionano data". Just wondering if this would make more sense to refer to this as data rather than molecules here and later in the paragraph.
Response:
Good point. We’re really using DNA molecules to create Bionano data (file names from Bionano are typically called things like ‘molecules.bnx’, so the ‘Bionano molecules’ term is reinforced by the naming convention of the files. We’ve change ‘Bionano molecules’ to ‘Bionano data’

Comment:
Irrelevant, but interesting: In table 4 for the reapr breaks for 10x, i wonder if these would vary much between the two pseudohap outputs...
Response:
I’d imagine that there might be some variation, but probably not enough to change the overall message.

Comment:

Discussion pg 28: You mention that you address heterozygosity, but that wasn't explicitly addressed in your analyses, so may just want to reword slightly or mention something about how much more (or not) the polecat is from the ferret or from the rat?
Response:
We've edited the sentence afterwards to start: "We address some of these factors…."

Comment:
Irrelevant also: I really am bummed that 10x assemblies wont be able to be generated for much longer because of the lawsuit. Idk if it would be worth mentioning that here or towards the end of the discussion, but it certainly makes your call to com up with new ways to do linked read assemblies more relevant...
Response:
Yes, I'm not sure if they'll introduce something similar to their Next GEM chip, or if someone else will take up the reigns. It's probably pushing the limits of the paper to discuss this though.

Comment:
General, supplement: Do you happen to have the pre-bionano tape station or agilent traces from the ferret? It might be interesting to show the difference of molecule dist sizes between that and the roadkill ferret sample.
Response:
Unfortunately, we didn't run a molecule size report on the ferret sample as we just went straight from the gel plug extraction to data generation.

Reviewer #2:
Comment:
For Supplementary Figure S3, the results shown are unsharp and the resolution of the picture needs to be increased.
Response:
The figures are in effect screen-grabs of the original data, as they are only available as embedded figures within a PDF file. In an attempt to make them clearer, we have created a new figure at higher resolution, where the results are 'stacked' one above the other instead of in a grid, so making them larger and easier to read.
