# Supplementary material for: Sequencing smart: De novo sequencing and assembly approaches for a non-model mammal
Source: Gigascience. 2020 May 12;9(5):giaa045. doi: 10.1093/gigascience/giaa045 (PMC7216774; doi:10.1093/gigascience/giaa045)
Supplement: giaa045_Supplemental_Files [file giaa045_supplemental_files.zip › Supplementary_Data.docx]

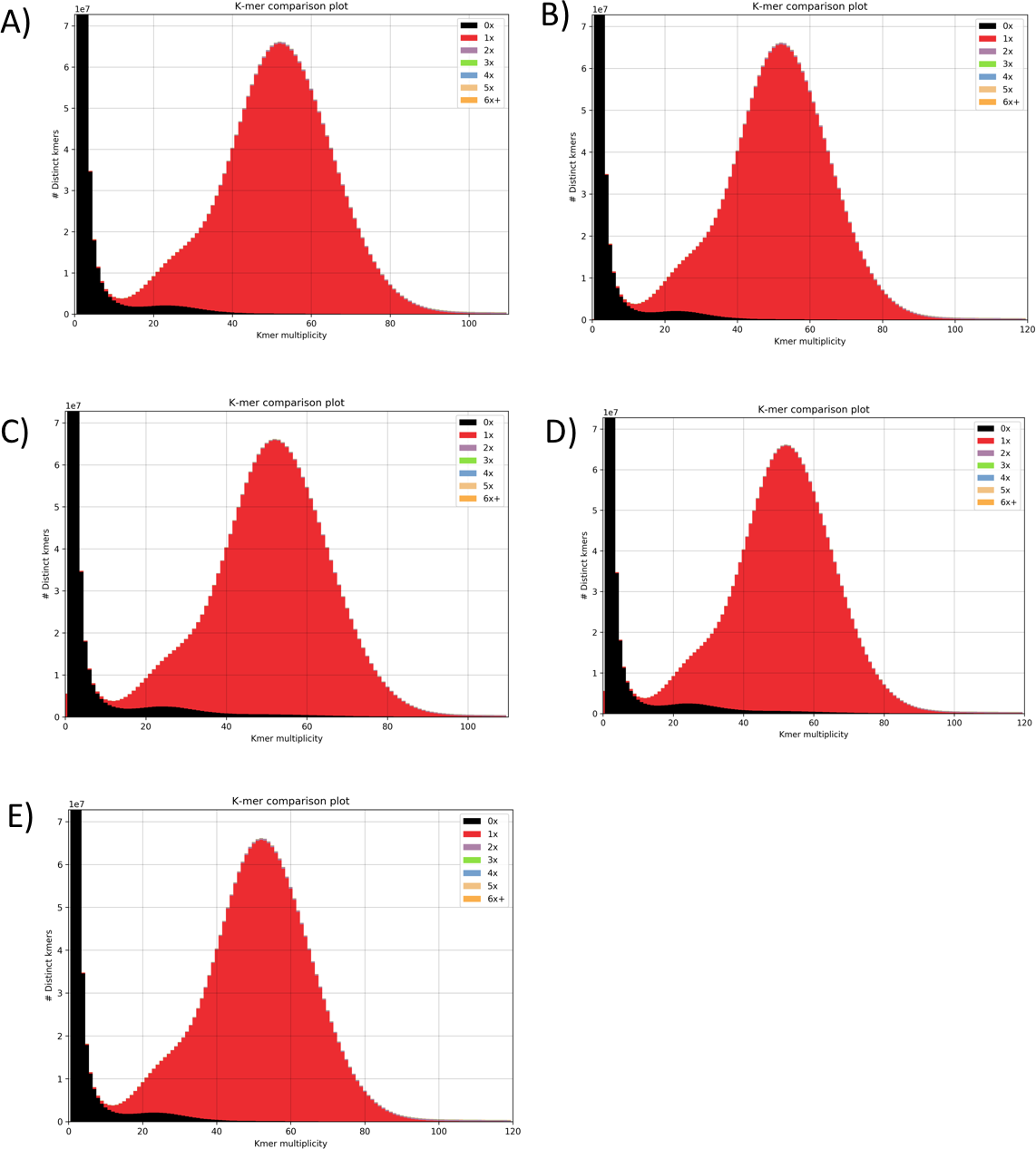


Figure S1. KAT k-mer plots comparing k-mer content of Illumina PCR-free reads with: A) w2rap assembly (A1), B) w2rap + lmp assembly (A2), C) 10x assembly (A3), D) 10x + lmp assembly (A4), and E) w2rap + 10x assembly (A9). The black area of the graphs represents the distribution of k-mers present in the reads but not in the assembly and the read area represents the distribution of k-mers present once in the reads and once in the assembly.


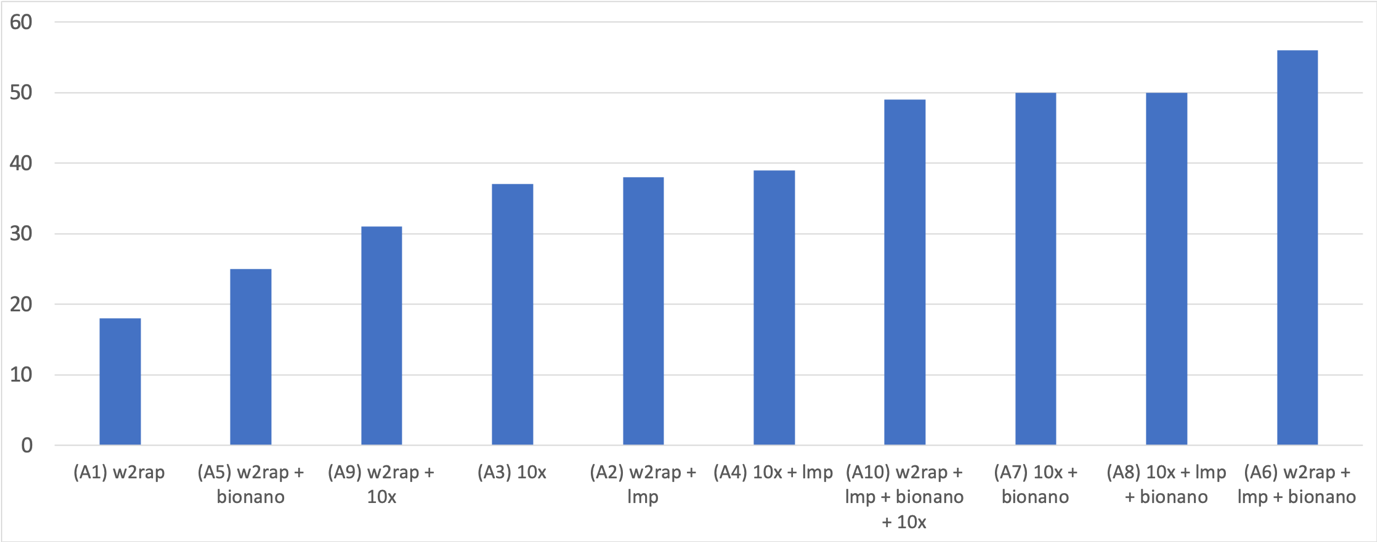


Figure S2. Rank-scoring for assemblies. Each assembly was ranked over seven key metrics. A rank-score of 10 was given to the highest-ranking assembly, down to a rank-score of 1 for the assembly that performed worst for the given metric.


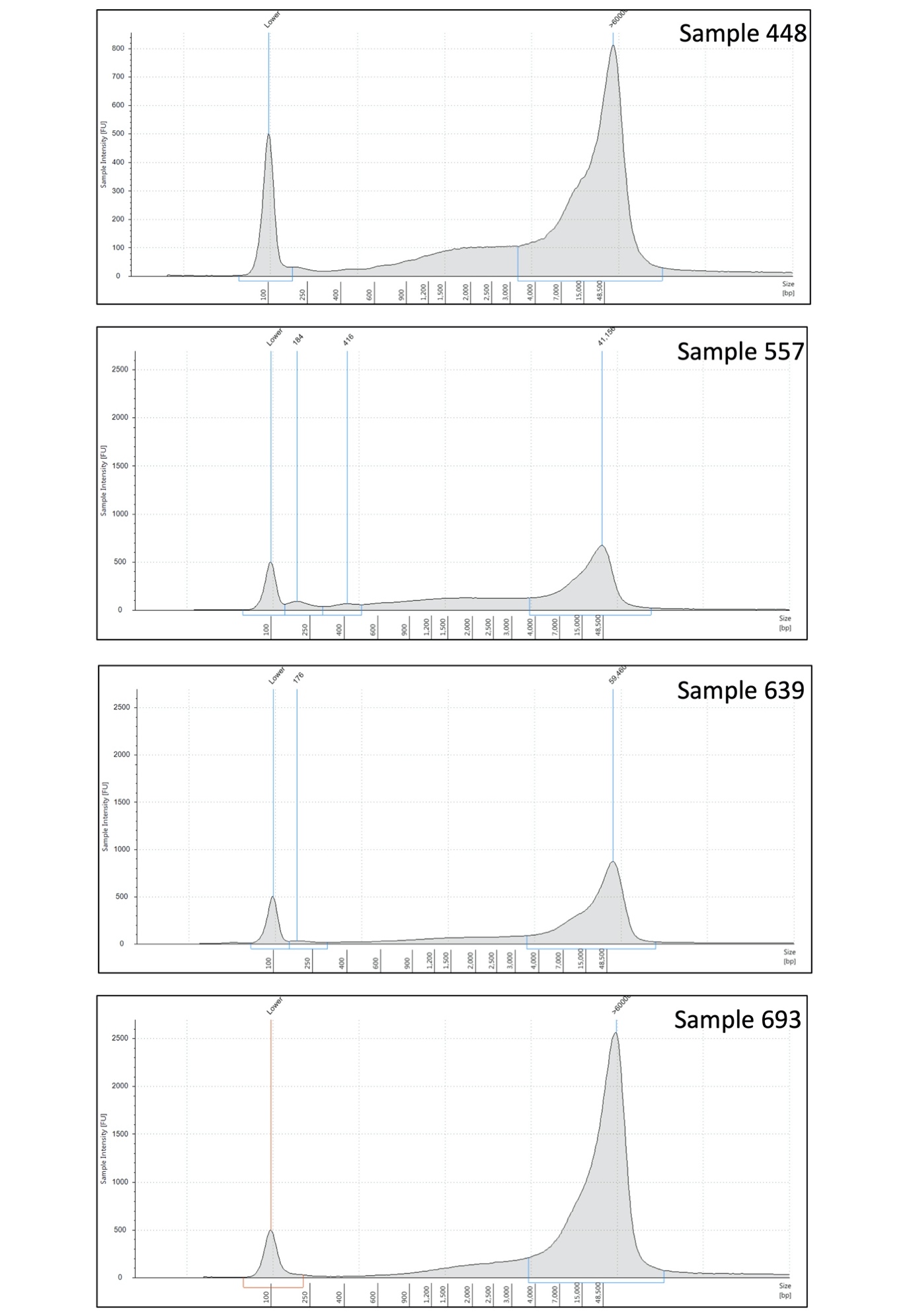


Figure S3. Agilent TapeStation traces for DNA extractions of VWT samples 448, 557, 639, and 693. The traces show the relative intensity of the number of molecules against the length of the molecules in base pairs.

| **Assembly number** | **Assembly short name** | **Data types** | **Assembly strategy and software** |
| --- | --- | --- | --- |
| **A1** | w2rap | PCR-free ISR | w2rap |
| **A2** | w2rap + lmp | PCR-free ISR, LMP | w2rap, SSPACE |
| **A3** | 10x | 10x Genomics | Supernova |
| **A4** | 10x + lmp | 10x Genomics, LMP | Supernova, SSPACE |
| **A5** | w2rap + bionano | PCR-free ISR, Bionano | w2rap, Bionano Solve |
| **A6** | w2rap + lmp + bionano | PCR-free ISR, LMP, Bionano | w2rap, SSPACE, Bionano Solve |
| **A7** | 10x + bionano | 10x Genomics, Bionano | Supernova, Bionano Solve |
| **A8** | 10x + lmp + bionano | 10x Genomics, LMP, Bionano | Supernova, SSPACE, Bionano Solve |
| **A9** | w2rap + 10x | PCR-free ISR, 10x Genomics | w2rap + scaff10x |
| **A10** | w2rap + lmp + bionano + 10x | PCR-free ISR, LMP, Bionano, 10x Genomics | w2rap, SSPACE, Bionano Solve, scaff10x |

Table S1. Ten different assembly strategies using a variety of different data types: PCR-free Illumina short-read (ISR), long mate-pair (LMP), 10x Genomics Chromium library, and Bionano Genomics optical maps.

Table S2 (see file Supplementary_Data_Table_S2.xlsx). Full assembly statistics for all assemblies.
