# Supplementary material for: Sequencing smart: De novo sequencing and assembly approaches for a non-model mammal
Source: Gigascience. 2020 May 12;9(5):giaa045. doi: 10.1093/gigascience/giaa045 (PMC7216774; doi:10.1093/gigascience/giaa045)
Supplement: giaa045_Response_to_Reviewer_Comments_Original_Submission [file giaa045_response_to_reviewer_comments_original_submission.pdf]

Author's Response To Reviewer Comments

Close

Please see personal cover document Response.docx

Close
