# Supplementary material for: Sequencing smart: De novo sequencing and assembly approaches for a non-model mammal
Source: Gigascience. 2020 May 12;9(5):giaa045. doi: 10.1093/gigascience/giaa045 (PMC7216774; doi:10.1093/gigascience/giaa045)
Supplement: giaa045_Reviewer_1_Report_Original_Submission — Ellie Armstrong -- 9/3/2019 Reviewed [file giaa045_reviewer_1_report_original_submission.pdf]

Reviewer Report

Title: Sequencing smart: De novo sequencing and assembly approaches for a non-model mammal.

Version: Original Submission **Date:** 9/3/2019

Reviewer name: Ellie Armstrong

Reviewer Comments to Author:

In general, I think the manuscript from Etherington et al. is an interesting exploration into the assembly of non-model organisms. The manuscript is in general well-written and thorough in explaining their analyses and do a thorough job of examining assembly errors in a variety of ways, which provides some much needed insight into the typical "plug and chug" genome assemblies being produced recently. In my opinion, I think it would be pertinent for the authors to provide a few additional analyses/results that I don't expect will be very time consuming, but I think would benefit the manuscript substantially. It is excellent to have another high-quality assembly completed for a non-model organism. I thoroughly enjoyed the manuscript. I have provided some overarching comments below, followed by line by line edits/suggestions.

First, I think because many of the author's arguments depend on their position that the polecat sample is low-quality, I think it would be helpful to include some of the DNA BioA traces or gel images to see the distribution of fragment lengths produced by the DNA extraction. It was not apparent to me why they chose either the length of MP insert sizes, nor the use of bionano technology over something like Hi-C, which is also very good at scaffolding. This should be addressed in some way and also would help the reader in understanding the limitations of any given technology.

Second, we have been getting quite high-quality assemblies from lower coverage 10x data (see Armstrong et al. 2018 for our take on this in african wild dogs--I am sure there are others, but obviously I know the most about this particular case). The 10x data presented here was sequenced at an extremely high coverage (85x) and no information is given on the molecule lengths output by the assembler. I think it would be worth it for the authors to test the assembler with a substantially lower coverage version of their dataset. If the assembly generated is comparable, this makes 10x a substantially cheaper technology to use than reported here. I suspect there will be some tradeoffs with this with lower-quality samples, but based on our experience, 30-40x data can still generate a good assembly.

Lastly, I think it would be relevant to add more motivation as to why the assemblers chosen were used. The data generated here would work well with the DISCOVAR (of which w2rap is quite similar, I believe) and ALLPATHS-LG. These assemblers are well known. If the team has the resources, it would be pertinent to test these as well because there are far more mammalian assemblies built with these software and it makes the results more comparable. However, I am aware that ALLPATHS-LG can be computationally very expensive for such a large genome and understand if this is not possible with the current resources.

I have provided line by line comments/suggestions below per section:

Introduction

Pg 3: "Adding more data to genome assemblies not always results" to "Adding more data to genome

assemblies does not always result"
Pg 4: I don't think you need the "Non-model organism header" the text seems to flow without the sub-headings in the introduction
Pg 4: May be useful to cite some recent efforts of de novo assembly and low coverage sequencing that have provided such insights?
Pg 5: "100kb being optimum" --I think this is a bit misleading. 100kb may be optimum (although not sure where that is stated from 10x--maybe just cite this?), but 50kb is enough to generate a high-quality assembly according to their page. You also mention this later in methods.
Pg 5: Sentence beginning with "Degraded DNA..." delete 'and' between 'populations' and 'is'.
Pg 5: Additionally think the subheader about the polecat is not needed
Pg 6: I think it would be worth it to discuss more (or cite evidence) of how much bias is incurred using PCR free or PCR methods for PE library prep. It was my understanding that at substantial coverage that bias is negligible in the resulting assembly. For non-models with low DNA quality, this would also mean you need as little as 1-5ng total rather than 2-5ug (which is a TON and not even possible from smaller species).
Pg 7: I may be entirely wrong, but I have never seen these referred to as LMP, and just MP...
Pg 7: Some contradictory statements on 10x here. It was my understanding that you require much, much less than this to prepare a 10x library...something like 1ng? so I am not sure where this is coming from. You also mention the dilution in your methods, so I am not sure where the 20ug/uL in 10uL is coming from, that is a lot of material!
Pg 8, Table 1: I think that it may be useful to refer to these as haplotigs rather than haplotypes. I would think that MP resolution (for example, if you prepare a 20kb insert library) can give you haplotig level information, depending on the length you are considering?
Materials & Methods
Pg 9: As I said above, the 10x data seems extremely high coverage to me.
Pg 10: May also be pertinent for the general audience to mention that w2rap is a modified version of DISCOVAR, which is a more well-known assembler
Pg 10: Why SSPACE? Why not something like ALLPATHS-LG etc?
Pg 10: Assembly A3 end of paragraph add "which creates one haplotype per scaffold at random"
Pg 10: Assembly A4, clarify what was used to scaffold. SSPACE again?
Pg 11: How was the Bionano data assembled?
Pg 12: "the number of scaffolds over given lengths" should be "the number of scaffolds of given lengths"
Pg 12: Why use the human chromosome as the reference? We know the karyotype of the polecat, so why not use something relevant to this species? The genome is substantially smaller and there are more chromosomes, so this may be skewing results.
Pg 13: May be useful to add in parameters used for RepeatMasker.
Pg 16 Table 2: It would be great to have some L90 statistics here or in the supplement, since this table is already quite large.
Pg 18: In the misassemblies section, it would be very interesting to see what scaffold sizes these breaks primarily occurred in and if they were in heterozygosity rich regions...
Pg 22 Figure 4: Might be good to explain why 2x + up is not visible on the plot, but is in the legend or do some sort of zoom if it is there, but not visible?

Pg 23: "probably down" should be "probably due"
Pg 23: I have no suggestion here other than it is bizarre and concerning to me that bionano created more fragmented orthologs...
Pg 25: Value for money section. If you do decide to add in addt'l assemblies using various data depths, it would be very interesting to see if these results change on the price points. If lower depth can be used/sequenced, this could shift substantially.
Pg 29: The scaffolding for the domestic ferret genome is not mentioned anywhere in methods. It may be worth adding a few sentences in and a table with full BUSCO scores and assembly stats (to supplementary, even)
Pg 30: Is prepping a 10x assembly not comparable with prepping mate-pair libraries? Especially if you are paying to have these services done, I have found that mate pair prep is more expensive even than the cost of a 10x library.
Pg 31: Here, I think when talking about sample quality it would be really useful to discuss your molecule length from your extractions.
Supplementary Methods
There is no information on tissue type, amount of tissue used or DNA extraction methods for any of these preps. This needs to be included, because different extraction protocols are suggested for 10x and bionano than for paired-end libraries.

Methods

Are the methods appropriate to the aims of the study, are they well described, and are necessary controls included? Choose an item.

Conclusions

Are the conclusions adequately supported by the data shown? Choose an item.

Reporting Standards

Does the manuscript adhere to the journal's guidelines on minimum standards of reporting? Choose an item.

Choose an item.

Statistics

Are you able to assess all statistics in the manuscript, including the appropriateness of statistical tests used? Choose an item.

Quality of Written English

Please indicate the quality of language in the manuscript: Choose an item.

Declaration of Competing Interests

Please complete a declaration of competing interests, considering the following questions:

- Have you in the past five years received reimbursements, fees, funding, or salary from an organisation that may in any way gain or lose financially from the publication of this manuscript, either now or in the future?
- Do you hold any stocks or shares in an organisation that may in any way gain or lose financially from the publication of this manuscript, either now or in the future?
- Do you hold or are you currently applying for any patents relating to the content of the manuscript?
- Have you received reimbursements, fees, funding, or salary from an organization that holds or has applied for patents relating to the content of the manuscript?
- Do you have any other financial competing interests?
- Do you have any non-financial competing interests in relation to this paper?

If you can answer no to all of the above, write 'I declare that I have no competing interests' below. If your reply is yes to any, please give details below.

I declare that I have no competing interests.

I agree to the open peer review policy of the journal. I understand that my name will be included on my report to the authors and, if the manuscript is accepted for publication, my named report including any attachments I upload will be posted on the website along with the authors' responses. I agree for my report to be made available under an Open Access Creative Commons CC-BY license (http://creativecommons.org/licenses/by/4.0/). I understand that any comments which I do not wish to be included in my named report can be included as confidential comments to the editors, which will not be published.

Choose an item.

To further support our reviewers, we have joined with Publons, where you can gain additional credit to further highlight your hard work (see: https://publons.com/journal/530/gigascience). On publication of this paper, your review will be automatically added to Publons, you can then choose whether or not to claim your Publons credit. I understand this statement.

Yes Choose an item.
