# Supplementary material for: Sequencing smart: De novo sequencing and assembly approaches for a non-model mammal
Source: Gigascience. 2020 May 12;9(5):giaa045. doi: 10.1093/gigascience/giaa045 (PMC7216774; doi:10.1093/gigascience/giaa045)
Supplement: giaa045_Reviewer_1_Report_Revision_1 — Ellie Armstrong -- 3/19/2020 Reviewed [file giaa045_reviewer_1_report_revision_1.pdf]

Reviewer Report

Title: Sequencing smart: De novo sequencing and assembly approaches for a non-model mammal.

Version: Revision 1 **Date:** 3/19/2020

Reviewer name: Ellie Armstrong

Reviewer Comments to Author:

Etherington et al. have responded well to all reviewer comments and have produced a very nice and thorough paper investigating various genome assembly technologies and their impact on degraded samples. The paper reads extremely well and I think the authors have done a particularly good job explaining some more advanced genome assembly concepts that I think will be very beneficial to those in the field dealing with non model organisms. I have included just some brief comments below that address some minor grammatical/sentence errors. I also hope the authors are doing well in this very uncertain time and wish them all the best with their future research. Great work to all!
Abstract, Background: "little is known about the correlation between genome sequencing..." I would just write relationship instead of correlation here. Correlation is a bit of a loaded word.
Introduction, top of pg 4: "the cost of generating this amount of data and assemble..." assemble should be "assembling"
Introduction, pg 5, paragraph 2: First sentence reads a bit funny saying that organisms are species from populations, maybe rephrase to: Many samples from non-model organisms originate from wild populations that are highly heterozygous"
Page 6 Sequencing technologies: "Recent machines" to just "Machines"
Page 7, Long Mate Pair sequencing: Clarification on this, is 4ug of DNA required to generate all 12 LMP libraries or is this per LMP library?
Materials and methods, page 9: "Using the same sample of a roadkill European Polecat sample"--delete second "sample", redundant
Materials and methods, page 9: You list the coverage for all the libraries except the four lanes of 150bp PE 10x, so maybe just add that in.
Page 15, Ranking assemblies: The first two sentences of this paragraph can probably be combined, seems repetative?
General comment: It might be useful in your tables and the text to add in ',' to your numbers. For example, 300,334 instead of 300334. I find that with a lot of big numbers it is easier to read this way and quickly get a sense for the magnitude of the difference, i.e. if its 1,000,000 vs 100,000 makes it easier to see that is larger than if you just have 1000000 vs 100000.
Materials and methods, page 9: "We generated 664 Gb of Bionano molecules" --just checking on how this is supposed to be referred to. Do you generate "Bionano molecules" or "Bionano data". Just wondering if this would make more sense to refer to this as data rather than molecules here and later in the paragraph.
Irrelevant, but interesting: In table 4 for the reapr breaks for 10x, i wonder if these would vary much between the two pseudohap outputs...

Discussion pg 28: You mention that you address heterozygosity, but that wasn't explicitly addressed in your analyses, so may just want to reword slightly or mention something about how much more (or not) the polecat is from the ferret or from the rat?
Irrelevant also: I really am bummed that 10x assemblies wont be able to be generated for much longer because of the lawsuit. Idk if it would be worth mentioning that here or towards the end of the discussion, but it certainly makes your call to com up with new ways to do linked read assemblies more relevant...
General, supplement: Do you happen to have the pre-bionano tape station or agilent traces from the ferret? It might be interesting to show the difference of molecule dist sizes between that and the roadkill ferret sample.

Methods

Are the methods appropriate to the aims of the study, are they well described, and are necessary controls included? Choose an item.

Conclusions

Are the conclusions adequately supported by the data shown? Choose an item.

Reporting Standards

Does the manuscript adhere to the journal's guidelines on minimum standards of reporting? Choose an item.

Choose an item.

Statistics

Are you able to assess all statistics in the manuscript, including the appropriateness of statistical tests used? Choose an item.

Quality of Written English

Please indicate the quality of language in the manuscript: Choose an item.

Declaration of Competing Interests

Please complete a declaration of competing interests, considering the following questions:

- Have you in the past five years received reimbursements, fees, funding, or salary from an organisation that may in any way gain or lose financially from the publication of this manuscript, either now or in the future?

- Do you hold any stocks or shares in an organisation that may in any way gain or lose financially from the publication of this manuscript, either now or in the future?
- Do you hold or are you currently applying for any patents relating to the content of the manuscript?
- Have you received reimbursements, fees, funding, or salary from an organization that holds or has applied for patents relating to the content of the manuscript?
- Do you have any other financial competing interests?
- Do you have any non-financial competing interests in relation to this paper?

If you can answer no to all of the above, write 'I declare that I have no competing interests' below. If your reply is yes to any, please give details below.

I declare that I have no competing interests.

I agree to the open peer review policy of the journal. I understand that my name will be included on my report to the authors and, if the manuscript is accepted for publication, my named report including any attachments I upload will be posted on the website along with the authors' responses. I agree for my report to be made available under an Open Access Creative Commons CC-BY license (http://creativecommons.org/licenses/by/4.0/). I understand that any comments which I do not wish to be included in my named report can be included as confidential comments to the editors, which will not be published.

Choose an item.

To further support our reviewers, we have joined with Publons, where you can gain additional credit to further highlight your hard work (see: https://publons.com/journal/530/gigascience). On publication of this paper, your review will be automatically added to Publons, you can then choose whether or not to claim your Publons credit. I understand this statement.

Yes Choose an item.
