# Supplementary material for: Sequencing smart: De novo sequencing and assembly approaches for a non-model mammal
Source: Gigascience. 2020 May 12;9(5):giaa045. doi: 10.1093/gigascience/giaa045 (PMC7216774; doi:10.1093/gigascience/giaa045)
Supplement: giaa045_Reviewer_2_Report_Original_Submission — Yu Jiang, Ph.D -- 12/26/2019 Reviewed [file giaa045_reviewer_2_report_original_submission.pdf]

**Reviewer Report**

**Title: Sequencing smart: De novo sequencing and assembly approaches for a non-model mammal.**

**Version: Original Submission** **Date:** 12/26/2019

**Reviewer name: Yu Jiang, Ph.D**

**Reviewer Comments to Author:**

In this paper the authors provide a reference for us to choose a platform and assembly strategy for obtaining sequence samples with lower quality or degradation. Additionally, the scaffold-level of the endangered European Polecat (Mustela putorius) genome was obtained through different sequencing data and assembly strategies, which can be valuable and potentially useful for protection of this species.
Major comments:
1. "non-model mammals" in the title of your article "Sequencing smart: De novo sequencing and assembly approaches for non-model mammals" is too extensive and exaggerated, could the European Polecat (Mustela putorius) fully represent non-model mammals? This can be easily misleading for researchers, and for the whole text you use "non-model mammals", I suggest to change to the European Polecat.
2. How do you evaluate and grade the "degraded and low-quality sample" in your manuscript and what are the detailed?
3. In the Sequencing paragraph of the Materials and Methods section, "Because the domestic ferret and its polecat ancestor diverged only around 2000 years ago, and fully interbreed we do not expect significant divergence and structural differences between the two species." Is there corresponding literature support? Otherwise, the corresponding evaluation results need to be given. This is an important reference for the rationality of using Bionano data to scaffold of the domestic ferret.
4. In the Discussion section, "Although chromosome-scale assemblies are now achievable, it is often not possible or necessary to assemble the genomes of non-model organisms to such precision." Hi-C sequencing technology is an important and widely used method for obtaining chromosome-scale assemblies, which is necessary for linkage-analysis in animal genomic studies such as QTL, WGAS and genome selection. Although it can be discussed from the perspective that low-quality samples cannot be sequenced for Hi-C or Bionano methods, but you did not evaluate the value of Hi-C, which has a major flaw in this work.
Minor comments:
1. In the Gene content paragraph of the Materials and Methods section, "For speed, 27 sequences that had tblastn runtimes of over 3 days were removed from the mammalia_odb9 database" why remove the 27 sequences that runtimes of over 3 days? I suggest you to use the latest BUSCO (v3.0.2) for verifying and finding out the real reason.
2. In the Repeats paragraph of the Results section, "RepeatMasker was used to look at Carnivora-specific repeat content in the assemblies." what is the version of RepeatMasker and the library?
3. In the Discussion section, the paragraph headings are bold or non-bold, and the formatting looks

confusing.

**Methods**

Are the methods appropriate to the aims of the study, are they well described, and are necessary controls included? Choose an item.

**Conclusions**

Are the conclusions adequately supported by the data shown? Choose an item.

**Reporting Standards**

Does the manuscript adhere to the journal's guidelines on [minimum standards of reporting?] Choose an item.

Choose an item.

**Statistics**

Are you able to assess all statistics in the manuscript, including the appropriateness of statistical tests used? Choose an item.

**Quality of Written English**

Please indicate the quality of language in the manuscript: Choose an item.

**Declaration of Competing Interests**

Please complete a declaration of competing interests, considering the following questions:

- Have you in the past five years received reimbursements, fees, funding, or salary from an organisation that may in any way gain or lose financially from the publication of this manuscript, either now or in the future?
- Do you hold any stocks or shares in an organisation that may in any way gain or lose financially from the publication of this manuscript, either now or in the future?
- Do you hold or are you currently applying for any patents relating to the content of the manuscript?
- Have you received reimbursements, fees, funding, or salary from an organization that holds or has applied for patents relating to the content of the manuscript?
- Do you have any other financial competing interests?
- Do you have any non-financial competing interests in relation to this paper?

If you can answer no to all of the above, write 'I declare that I have no competing interests' below. If your reply is yes to any, please give details below.

I declare that I have no competing interests

I agree to the open peer review policy of the journal. I understand that my name will be included on my report to the authors and, if the manuscript is accepted for publication, my named report including any attachments I upload will be posted on the website along with the authors' responses. I agree for my report to be made available under an Open Access Creative Commons CC-BY license (http://creativecommons.org/licenses/by/4.0/). I understand that any comments which I do not wish to be included in my named report can be included as confidential comments to the editors, which will not be published.

Choose an item.

To further support our reviewers, we have joined with Publons, where you can gain additional credit to further highlight your hard work (see: https://publons.com/journal/530/gigascience). On publication of this paper, your review will be automatically added to Publons, you can then choose whether or not to claim your Publons credit. I understand this statement.

Yes Choose an item.
